# Green Solid-State Synthesis of Antibacterial Binary Organic Material: Crystal Growth, Physicochemical Properties, Thermal Study, Antibacterial Activity, and Hirshfeld Surface Analysis

**DOI:** 10.3390/ijms26125509

**Published:** 2025-06-09

**Authors:** Adarsh Rai, Sumit Chaudhary, Surya Prakash Dube, Szymon Bajda, Richa Raghuwanshi, Shiva Kant Mishra, Gaetano Palumbo, Rama Nand Rai

**Affiliations:** 1 Department of Chemistry, Institute of Science, Banaras Hindu University, Varanasi 221005, India; rai@agh.edu.pl (A.R.); sumitchaudhary.sam@gmail.com (S.C.); 2 Faculty of Metals Engineering and Industrial Computer Science, AGH University of Krakow, Mickiewicza 30, 30-059 Krakow, Poland; 3 Department of Botany, MMV, Banaras Hindu University, Varanasi 221005, India; suryadubey@bhu.ac.in (S.P.D.); richabhu@yahoo.co.in (R.R.); 4 Department of Materials Science and Engineering, Indian Institute of Technology, Kanpur 208016, India; skant@iitk.ac.in; 5 Faculty of Foundry Engineering, AGH University of Krakow, Mickiewicza 30, 30-059 Krakow, Poland; gpalumbo@agh.edu.pl

**Keywords:** phase diagram, solid-state synthesis, intermolecular compound, single crystal, antibacterial activity

## Abstract

The organic compounds 2-aminopyrimidine (AP) and 4-aminobenzoic acid (PABA) were selected for the synthesis of a compound by establishing the phase diagram and adopting the solid-state synthesis method. The phase diagram analysis suggested the formation of a novel intermolecular compound (IMC) at a 1:1 stoichiometric ratio of AP and PABA, along with two eutectics at 0.25 and 0.90 mole fractions of AP. FTIR and NMR spectroscopy were used for the structure elucidation of the intermolecular compound. The powder X-ray diffraction analysis revealed the novel nature of IMC (APPABA) and the mechanical mixture nature of eutectics. The sharp and single peak of the DSC curve suggested the melting and pure nature of the synthesized IMC. Various thermodynamic parameters of IMC and eutectics were studied. A single crystal of the IMC was grown from solution and its single-crystal X-ray diffraction analysis revealed that it crystallized in a monoclinic system with the P21/n space group. Hirshfeld surface analysis further validated the weak non-covalent interactions summarized through the single-crystal X-ray analysis. Studies on the IMC were thoroughly conducted to evaluate its antibacterial activity with reference to antibiotics, and it showed significant positive responses against various pathogenic microbial isolates (*Staphylococcus aureus*, *Escherichia coli*, *Pseudomonas aeruginosa*, *Klebsiella aerogenes*, and *Shigella boydii*) and non-pathogenic microbial isolates (*Enterobacter cloacae*, *Pseudomonas azotoformans*, and *Burkholderia paludis*). It was also found effective against methicillin-resistant bacterial strains viz. *Staphylococcus aureus* MRSA.

## 1. Introduction

**Figure 1:**
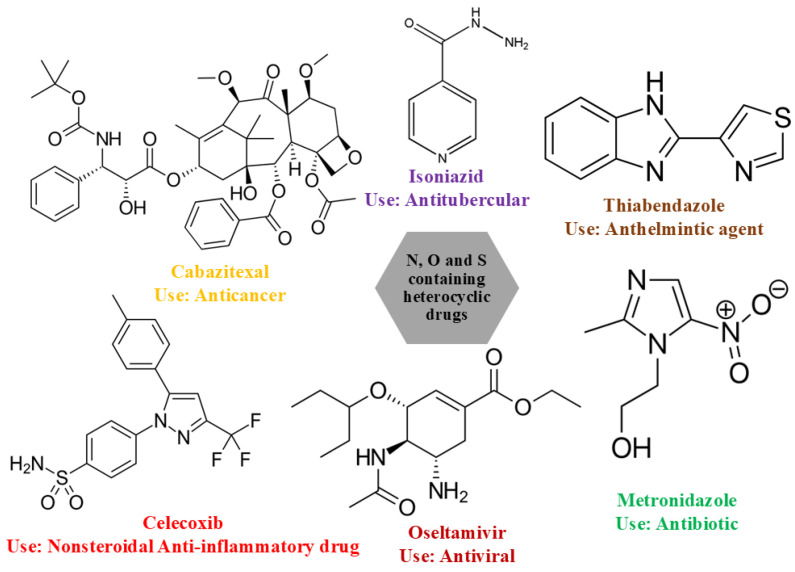
Chemical structure of drugs containing heteroatom and its biological activity.

Over the years, the world has witnessed several important applications of organic compounds, which are of immense importance, particularly in the field of biomedical and pharmaceutical sciences as biomaterials [1,2]. International health organizations and research scientists across the globe are serious about the infectious diseases which are posing a mortal threat to human health. The rise in health hazards and excessive use of antibiotics influence the pathogens to become resistant to them. The development of newer and more advanced drugs is demanded to work effectively against both drug-sensitive and drug-resistant bacteria [3]. There are a number of ways by which advanced drugs can combat both drug-sensitive and drug-resistant bacteria by leveraging structural modifications and enhanced delivery mechanisms [4,5,6]. Additionally, the parent compounds, AP and PABA, are known for various biological applications. Although they inherently exhibit drug resistance, their analogs or derivatives are being investigated for their potential to overcome this resistance [7,8]. In this regard, the combination of multi-drug therapy is now taken into consideration for the treatment of cancer, HIV, malaria, etc. The organic ligands, containing heteroatoms such as nitrogen, sulphur, and oxygen in the aromatic ring system (Figure 1), often possess important medicinal properties such as antibiotic [9], anti-cancer [10], antitubercular [11], anthelmintic, antiviral [12,13], and anti-inflammatory [14]. Nonetheless, the solubility and bioavailability of the synthesized drug molecules are major challenges in the pharmaceutical sector. To improve and overcome these issues, binary organic materials such as eutectics [15], deep eutectics [16], co-crystals [17] and salts formed through salt formation [18] have been recognized in the past few years. Systematic studies on binary organic materials, focusing on their physicochemical properties, thermodynamic behaviour, solidification behaviour, thermal stability, reaction kinetics, hygroscopicity, and solubility [19,20,21,22,23], provide valuable parameters for evaluating newer organic materials that exhibit improved performance compared to that of the starting parent compounds.

The adopted solid-state green synthesis approach is significant because it is environmentally friendly, solvent-free, non-tedious, less time-consuming and cost-effective, compared to many other synthetic procedures. The study of the phase diagram reveals the nature of each binary composition, solidification behaviour, and phases that are in equilibrium at specific temperatures and compositions. It also reveals the stoichiometric ratio of parent compounds that could yield the novel material with up to 100% efficiency. The solid-state synthesis helps in avoiding the use of solvents. Because of the importance of binary organic materials, two starting parent compounds, 2-aminopyrimidine (AP) and 4-aminobenzoic acid (PABA) are selected to search the materials with medicinal properties. The nitrogen-containing heterocyclic ring, such as pyrimidine, is the key structural unit in many natural and synthetic biomolecules that are known for their significant applications in medicinal chemistry [24,25]. 2-aminopyrimidine and its derivatives have special significance among the biologically active classes of compounds [26] that are known for a wide spectrum of such as antimicrobial [27,28], anti-oxidant [24], lysine-specific demethylase-1 (LSD1) inhibition [29], anti-inflammatory [28], antitubercular [30], adenosine receptor antagonism [31], antitumor [32], and antimalarial activity [33]. The organic compound 4-aminobenzoic acid is a member of the Vitamin B complex and has been known as an antibacterial agent [34].

In view of the significant importance of the parent compounds, AP and PABA, they were selected for the detailed study of the phase diagram, the formation of IMC, eutectics, and systematic investigation of their thermal behaviour, thermal properties, and structural changes. The synthesized intermolecular compound (APPABA) was characterized using differential scanning calorimetry (DSC), FTIR, NMR, X-ray diffraction, UV–vis absorption and fluorescence emission properties. The single crystal of the novel intermolecular compound was grown, and the crystal of IMC was studied to determine its crystal structure and atomic packing. The IMC was also thoroughly investigated to assess its biological activity against non-pathogenic and pathogenic bacteria.

## 2. Results and Discussion

### 2.1. Phase Diagram Analysis

**Figure 2:**
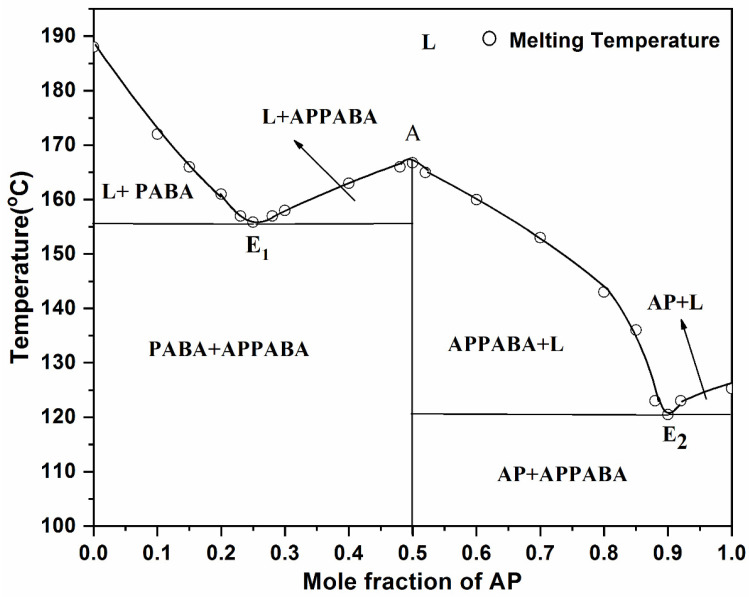
Phase diagram of the AP—PABA system.

The phase diagram between 2-aminopyrimidine (AP) and 4-aminobenzoic acid (PABA), is presented in Figure 2 in terms of the composition–temperature curve, where the mole fraction of the parent component (AP) is plotted on the *X*-axis and the corresponding melting temperature on the *Y*-axis. The phase diagram suggests that with the addition of AP to PABA, i.e., as the mole fraction of PABA decreases, the melting temperature of PABA in the mixture decreases and continues to decrease until it reaches the first lowest melting temperature, 167 °C, at point E_1_, which is the first eutectic point at a 0.25 mole fraction of AP. From this point, the further increase in the mole fraction of AP causes the increase in the melting temperature of the mixture and reaches the maximum melting temperature (156 °C) at point A, which indicates the formation of a new and stable intermolecular compound. Point A is a congruent melting point where the compositions of the solid and the melt are the same. The further increase in the mole fraction of AP again causes a decrease in the melting temperature of the mixture, which reaches the second minimum point (120 °C) at E_2_, which is the second eutectic of the system at a 0.90 mole fraction of AP. It is clearly obvious that the resulting intermolecular compound plays the role of one of the parent components of both eutectics.

At E_1_ and E_2_, the respective eutectic reaction may be represented as follows:

E_1_, L ⇌ + S_PABA_ + S_IMC_ (1)

E_2_, L ⇌ + S_IMC_ + S_AP_ (2)

The above equations represent the reaction occurring at the eutectic points (E_1_ and E_2_), where the homogeneous liquid phase is in equilibrium with two solid phases, and the points E_1_ and E_2_ are invariant points with zero degrees of freedom. The experimental solid–liquid equilibrium data and liquid mole fractions X_AP_ for the AP—PABA system have been provided in the Supplementary Materials (Table S1). Additionally, the phase diagram indicates that the formation of hydrogen bonds between the parent compounds may be possible, as the melting temperature of the IMC increases while remaining lower than that of PABA (Figure S1) [35]. Furthermore, hydrogen bonding significantly influences the growth of larger-sized crystals, as discussed in Section 2.3.2. The extent of hydrogen bonding in all three directions leads to stronger interactions among the molecules, resulting in 3D crystal packing [36,37].

### 2.2. Spectral Studies

#### 2.2.1. FTIR Absorption Studies

The FTIR studies suggest the formation of the intermolecular compound and give information regarding the functional groups that are involved in the interaction between the parent components. In the case of 2-aminopyrimidine (AP), the peaks observed at 3353 cm^−1^ and 3171 cm^−1^ are due to the asymmetric and symmetric stretching vibration of the primary amine group (–NH_2_) attached to the aromatic ring. The peak observed at 1041 cm^−1^ is due to aliphatic C–N stretching vibrations, the peaks observed at 1132 cm^−1^ and 1179 cm^−1^ are due to aromatic C–N stretching vibrations, while the peak at 999 cm^−1^ is attributed to NH_2_ out-of-plane bending vibrations. In the case of 4-aminobenzoic acid (PABA), the peaks observed at 3382 cm^−1^ and 3364 cm^−1^ are due to the asymmetric and symmetric stretch of N–H. The peak due to the free –OH group is observed at 3461 cm^−1^. The characteristic carbonyl (C=O) peak is observed at 1660 cm^−1^. The peak observed at 1292 cm^−1^ is due to C–N stretching, while the peaks observed at 1422 cm^−1^ and 964 cm^−1^ are due to O–H bending vibrations. The CH_2_ bending vibration observed at 843 cm^−1^ corresponds to the 1–4 substitution.

**Figure 3:**
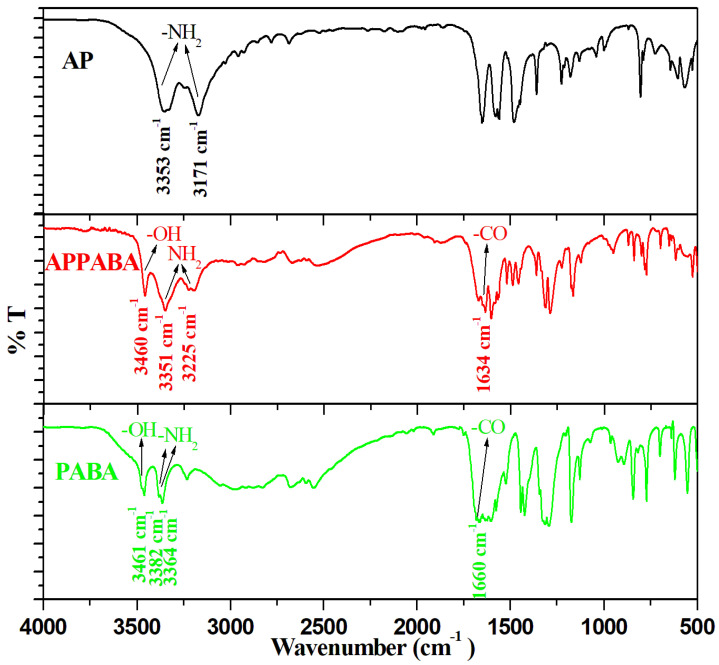
FTIR spectra of AP, PABA and the APPABA compound.

The FTIR spectrum of the intermolecular compound (APPABA) suggests certain differences compared to the FTIR spectra of the parent components (Figure 3). There are significant changes in the stretching frequency of NH_2_ of AP and OH of PABA in the intermolecular compound. Interestingly, the NH_2_ stretching vibrations of PABA could not be observed in the intermolecular compound, while a shift to a lower wave number in the carbonyl group of PABA from 1660 cm^−1^ to 1634 cm^−1^ could also be observed. All these changes in the vibration frequencies of functional groups suggest their involvement in hydrogen bonding among the parent compounds to yield the new intermolecular compound.

#### 2.2.2. NMR Studies

NMR spectrum of 2-aminopyrimidine (AP)

The proton NMR spectrum of 2-aminopyrimidine shows three major signals. The signal of the amino protons (–NH_2_) appears as a singlet at 6.53 ppm. The signal for the H_a_ proton appears as a triplet at 6.48 ppm, while the signal for the H_b_ proton appears as a doublet at 8.20 ppm. The carbon NMR spectrum of 2-aminopyrimidine shows three signals: the signal at 164.05 ppm is due to C_1_ carbon, C_2_ and C_4_ are equivalent carbons, and their signal appears at 158.06 ppm, while the signal for C_3_ carbon appears at 110.29 ppm. The ^1^H and ^13^C NMR spectra are shown in Figure S2 (Supporting Information).

NMR spectrum of 4-aminobenzoic acid (PABA)

The proton NMR spectrum of PABA shows four signals. The signal due to carboxylic proton appears at 11.95 ppm, while the amino protons appear at 5.87 ppm. The signals appearing at 7.62 ppm and 6.54 ppm are due to the aromatic ring protons. The carbon NMR spectrum of PABA shows five signals. The signal due to the carbonyl carbon (C_1_) appears at 168.04 ppm, whereas the signals for other carbon atoms appear at 117.37 ppm for C_2_, 131.57 ppm for C_3_, 112.73 ppm for C_4_, and 153.42 ppm for C_5_ carbon, respectively. The ^1^H and ^13^C NMR spectra are shown in Figure S3.

NMR spectrum of intermolecular compound (APPABA)

The synthesized IMC was soluble in polar solvents such as ethanol, water, acetone, methanol, and DMSO. For the NMR analysis, DMSO-d6 was used as the solvent. The proton NMR of the synthesized intermolecular compound (APPABA) shows five signals, which are present in either of the parent compounds (AP and PABA) at 8.22 ppm, 7.62 ppm, 6.60 ppm, 6.55 ppm, and 5.84 ppm. However, the signal due to the carboxylic proton present in PABA (11.95 ppm), as well as the signal due to the amino proton present in AP (6.53 ppm), were completely missing in the synthesized intermolecular compound, which suggests that there might be some intermolecular hydrogen bonding interactions involved via these functional groups among the parent compounds. The ^1^H and ^13^C NMR spectra of APPABA are given in Figure S4.

#### 2.2.3. Mass Spectral Analysis

The calculated *m*/*z* [M]+ values of the parent compounds (AP and PABA) and the synthesized novel compound APPABA were found to be 95.10, 137.138, and 232.238, respectively. However, the mass spectrum showed values of 96.054, 138.053, and 232.891, respectively. These observations confirm the formation of the APPABA compound and also support the findings of the NMR studies. The recorded mass spectra of the parent compounds and the synthesized APPABA are provided in Supplementary Figures S5–S7.

### 2.3. X-Ray Diffraction Studies

#### 2.3.1. Powder X-Ray Diffraction

**Figure 4:**
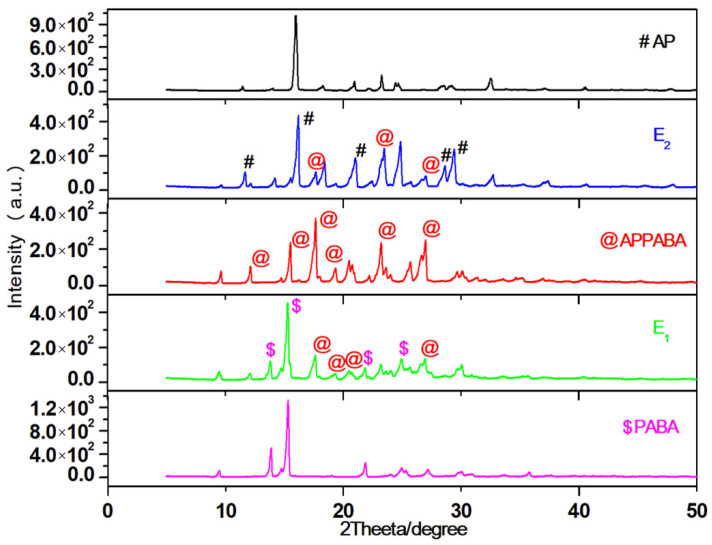
PXRD patterns of AP, PABA, eutectics (E_1_ and E_2_), and the APPABA intermolecular compound.

The powder X-ray diffraction (PXRD) pattern of parents, AP and PABA, eutectics, and the IMC are depicted in Figure 4. The Bragg peaks of AP, PABA, and the intermolecular compound (APPABA) are assigned, respectively, using the #, $ and @ symbols. In the PXRD pattern of the intermolecular compound (APPABA), some distinct new peaks are observed, which could not be assigned for any of the parent compounds, thereby suggesting that the IMC is a new entity. However, in the case of the eutectics (E_1_ and E_2_), the PXRD pattern of eutectic (E_1_) is a blend of the peaks of PABA and APPABA, while the PXRD pattern of eutectic (E_2_) is the blend of AP and APPABA. These observations obtained from the PXRD pattern of the eutectics (E_1_ and E_2_) suggest that they are not new entities, but rather simple mechanical mixtures of either the parent compounds or the intermolecular compound.

#### 2.3.2. Single-Crystal Growth and Single-Crystal Diffraction

**Figure 5:**
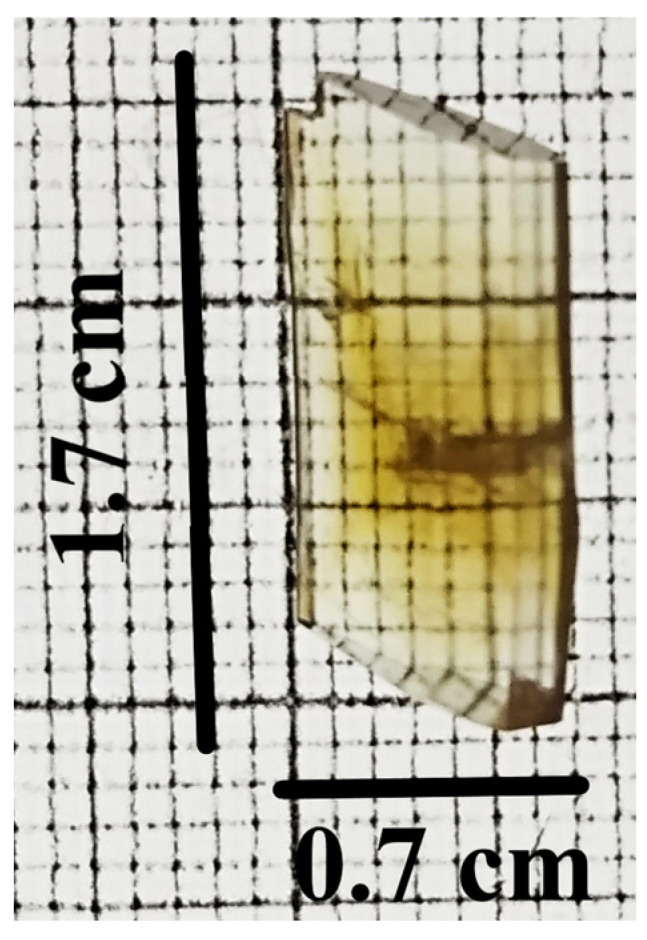
Photograph of grown single crystal of APPABA intermolecular compound, measuring 1.7 cm × 0.7 cm.

**Figure 6:**
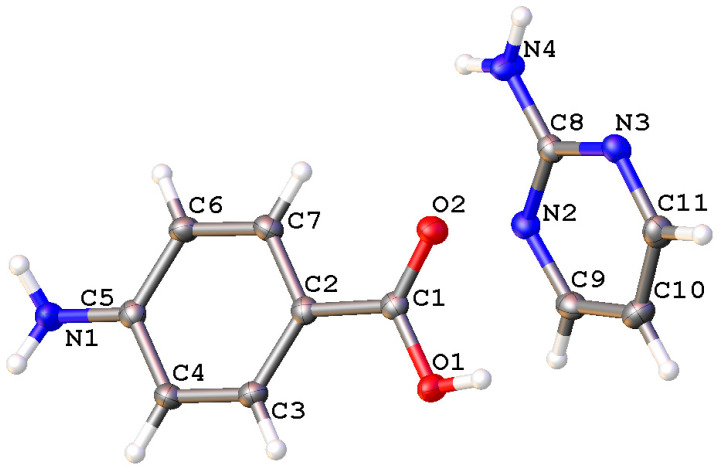
Molecular structure of the intermolecular compound (APPABA).

**Figure 7:**
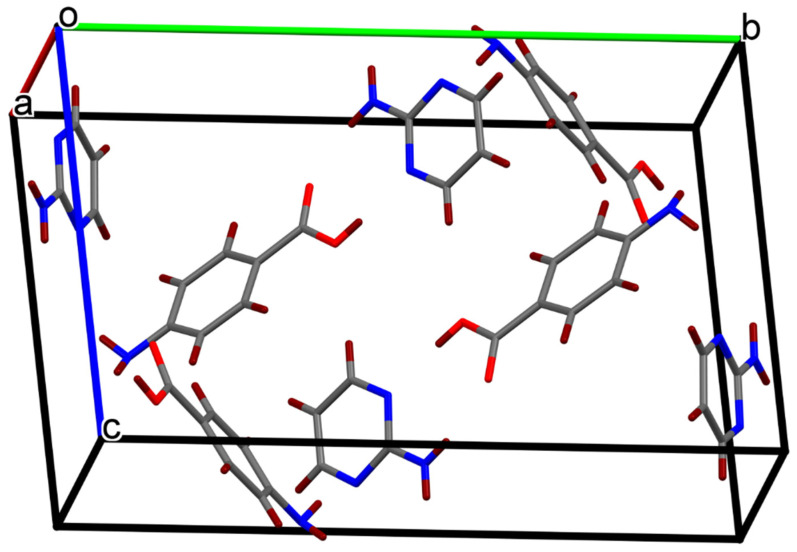
Packing diagram of the intermolecular compound (APPABA).

**Figure 8:**
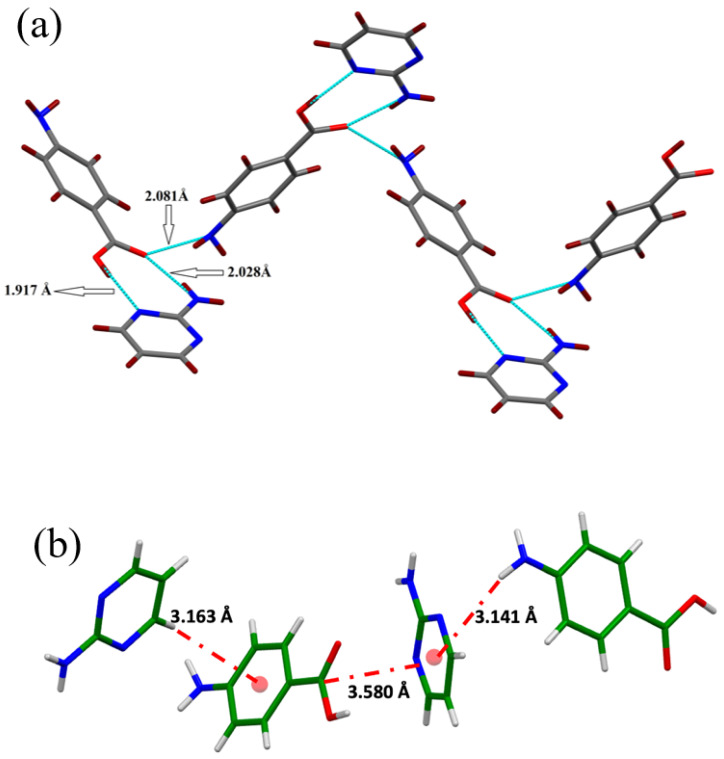
(**a**) Zigzag structure due to hydrogen bonding in the crystal along the c-axis and (**b**) non-covalent interactions in the synthesized novel compound (APPABA).

The single crystals of the newly synthesized compound APPABA were grown from the mixed solvent using the slow evaporation technique. A photograph of one of the grown APPABA crystals with dimensions of 1.7 cm (length) and 0.7 cm (width) is shown in Figure 5. A small piece was cut from the large-sized crystal to perform the single-crystal X-ray diffraction and crystal analysis. The solved crystal structure of APPABA and its ORTEP diagram (Figure 6) reveal that the synthesized APPABA compound is a co-crystal of its parent compounds, 4-aminobenzoic acid (molecule-A) and 2-aminopyrimidine (molecule-B). The APPABA crystallized in the monoclinic P21/n space group, with both molecules in the asymmetric unit and two molecules in the unit cell. The important crystallographic parameters and additional details are tabulated in Table 1. Molecule A and molecule B are joined together via hydrogen bonding between the carboxylic group of molecule A with the amine group and ring nitrogen atom of molecule B, i.e., O=C–O–H-----N and OH–C=O-----H–N–H. The packing diagram of the co-crystal APPABA is given in Figure 7. When viewed along the c-axis, the growth pattern of the co-crystal reveals a zigzag-type structure (Figure 8a). The crystal structure of APPABA reveals the non-covalent C-H---π and N-H---π interactions, as depicted in Figure 8b. The red dashed lines represent weak hydrogen bonding interactions between C–H and aromatic π-systems (C-H–π interactions) at a distance of 3.163 Å. The C-π interactions at 3.580 Å, along with N–H and the aromatic π-system (NH–π interaction) at 3.141 Å are observed. However, the novel crystal does not contain any π—π stacking.

#### 2.3.3. Hirshfeld Surface Analysis

**Figure 9:**
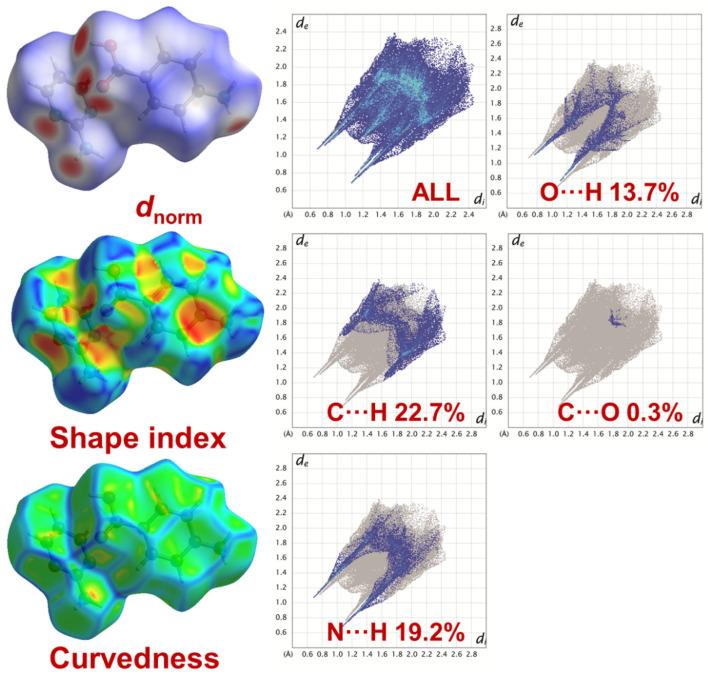
Hirshfeld field surfaces (d_norm_, shape index, and curvedness, respectively) of the APPABA intermolecular compound.

The Hirshfeld surfaces of APPABA are presented in Figure 9. The *d*_norm_ surfaces for both clusters are mapped over a *d*_norm_ range of −0.5 to 1.5 Å. In addition, the surfaces are presented as transparent for the visualization of the aromatic and puckered ring moieties around which they were computed. The weak non-covalent interactions discussed in the X-ray crystallography section are summarized effectively as the deep red circular depressions in the *d*_norm_ surfaces, indicating strong non-covalent interactions. The dominant C∙∙∙H, O∙∙∙H, N∙∙∙H, and C∙∙∙O interactions in APPABA exist in Hirshfeld surface plots as the red shaded area. Additionally, the small area and light colour on the surface represent weaker and longer contacts.

In addition, in the fingerprint plots of the intermolecular compound, APABA, the complementary regions can be visualized, where one molecule acts as a donor (d_e_ > d_i_) and the other as an acceptor (d_e_ < d_i_). In the IMC, the C∙∙∙H interaction appears in the region 1.6 Å < (d_e_ + d_i_) < 2.4 Å, contributing 22.7%, represented as a butterfly-shaped pattern; the O∙∙∙H intermolecular interaction appears within the range 1.1 < (d_e_ + d_i_) < 2.3 Å, contributing 13.7%, represented as a sharp pair of spikes; the N∙∙∙H intermolecular interaction appears within the range 1.0 Å < (d_e_ + d_i_) < 2.4 Å, contributing 19.2%, represented as a pair of spikes connected at the ends; and the C∙∙∙O intermolecular interaction appears at mid portion of the fingerprint plot, within the range 1.8 Å < (d_e_ + d_i_) < 2.0 Å, contributing 0.3% to the full fingerprint 2D plots.

### 2.4. Thermochemistry and Thermodynamic Studies

#### 2.4.1. Differential Scanning Calorimetry and Thermal Studies

**Figure 10:**
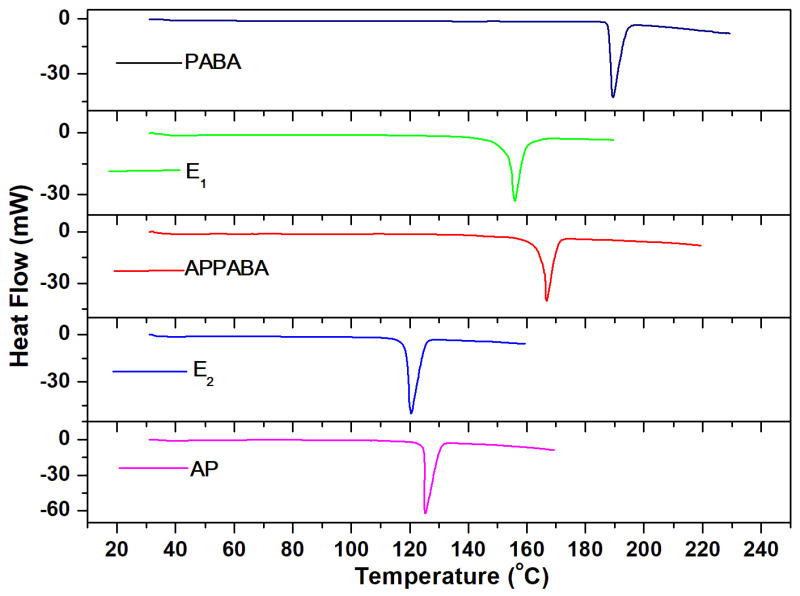
DSC thermogram of AP, PABA, eutectics (E_1_ and E_2_), and the APPABA compound.

The thermal behaviour and properties of the synthesized intermolecular compound and eutectics have been studied to get an idea about the thermal stability, phase transitions as well as the nature of interaction existing between the parent compounds. The differential scanning calorimetry (DSC) curves depicted in Figure 10 show the melting peaks of both eutectics and a sharp melting peak for the intermolecular compound, similar to the parent compounds (AP and PABA). The sharp and single melting peak of APPABA suggests its pure nature. The experimental heat of fusion values obtained from the DSC curves, along with the theoretically calculated heat of fusion values, using mixture law [38], are reported in Table 2. The heat of mixing values have been estimated, which helps in understanding the association behaviour of molecules in the eutectic melt. Based on the heat of mixing values, three types of structures are possible [39]. However, the study of interfacial energy, Jackson’s roughness parameter, and grain boundaries is important, as these parameters significantly influence crystal growth and crystal morphology. The interfacial energies were calculated using the formula [40]:

(3) $$\sigma =\frac{{C\Delta }_{fus}H}{({N_{A})}^{\frac{1}{3}}({V_{m})}^{\frac{2}{3}}}$$

where N_A_ is the Avogadro number, *V_m_* is the molar volume, and parameter C is constant, and it lies between 0.30 and 0.35. The value of C used for calculation was 0.35. Jackson’s roughness parameter was studied using the following relation:

(4) $$\alpha =\frac{{\xi \Delta }_{fus}H}{RT}$$

where ξ is a crystallographic factor that is generally less than or equal to one; for the present calculation, it is taken as 1. The interfacial energy and roughness parameter are tabulated in Table 3.

#### 2.4.2. Excess Thermodynamic Function

The excess thermodynamic functions signify the deviation from the ideal behaviour, in terms of excess free energy (g^E^), excess enthalpy (h^E^), and excess entropy (s^E^). These excess thermodynamic parameters were studied using the methods reported earlier [40], and the calculated values thus obtained are tabulated in Table 4.

### 2.5. Optical Studies

#### 2.5.1. UV–Vis Absorption Studies

**Figure 11:**
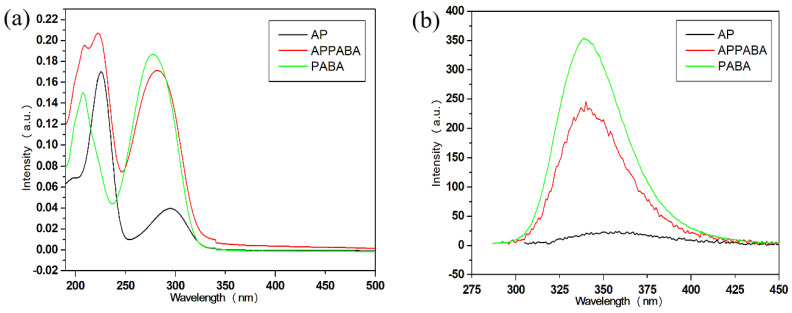
(**a**) UV–vis absorption and (**b**) emission spectra of AP, PABA, and the intermolecular compound APPABA.

To study the optical property, the UV–vis absorption spectra of the intermolecular compound (APPABA) and the parent compounds (AP and PABA) were recorded at room temperature in a methanol solution within the wavelength range of 190–1200 nm, and the spectra are shown in Figure 11a. Since n→π* transitions occur at longer wavelengths, while π→π* transitions take place at shorter wavelengths [41], the absorption spectrum of AP shows two bands: one at 225 nm, corresponding to the π→π* transitions, and the other at 295 nm, associated with the n→π* transitions. The other parent compound, PABA, also shows two absorption bands (208 nm and 277 nm). The band at 208 nm is ascribed to π→π* while the band at 277 nm is ascribed to n→π* (primary amino group) transitions. The UV–vis spectrum of the intermolecular compound (APPABA) shows three absorption bands (209 nm, 223 nm and 282 nm). It is evident from Figure 11a that the absorption bands in the intermolecular compound, occurring at 209 nm and 223 nm, are of higher intensity compared to those of both parent compounds. The band observed at the maximum wavelength in the intermolecular compound is found to be red-shifted and hypsochromic compared to PABA, while it is blue-shifted and hyperchromic compared to AP. These changes in the absorption spectra suggest the formation of an intermolecular compound (APPABA).

#### 2.5.2. Emission Studies

To study the fluorescence emission properties and compare the fluorescence of the synthesized APPABA to that of the parent compounds (AP and PABA), the same solution of each sample was used under identical conditions; however, each sample was excited with their respective maximum absorption wavelength. The experimental observation (Figure 11b) reveals that one of the parent compounds, AP, does not show any notable fluorescence emission, whereas the other parent compound, PABA, shows highly intense fluorescence emission. However, the intermolecular compound, APPABA, when excited with its maximum wavelength (λ_max_, 282 nm), exhibits stronger fluorescence emission than one of its parent compounds (AP) but less fluorescence emission than another parent compound (PABA) at 340 nm with a Stoke shift of 58 nm.

### 2.6. Antibacterial Activity

The inhibitory activity of the synthesized intermolecular compound, APPABA, was evaluated with reference to antibiotics against different bacterial isolates cultured on yeast extract mannitol agar (YEMA) medium. The activity was determined in terms of the diameter of the inhibition zone formed around the disc by the diffusion of APPABA and antibiotics viz. chloramphenicol, ampicillin, and tetracycline [42]. The different concentrations (100, 10, 1, and 0.1 µg/mL in DMSO) of antibiotics and the synthesized compound were obtained through serial dilutions. The zone of clearance formed by APPABA and antibiotics around the disc indicated positive antibacterial activity. The non-pathogenic bacteria isolated from field soil in Eastern UP, India, were previously characterized [43] and assigned with GenBank Accession No. *Enterobacter cloacae* (MK501756.1), *Pseudomonas azotoformans* (MK500938.1), and *Burkholderia paludis* (MK439528.1). The pathogenic bacteria (*Staphylococcus aureus* ATCC 29213, *Staphylococcus aureus MRSA* ATCC 43300, *Escherichia coli* ATCC 25922, *Pseudomonas aeruginosa* ATCC 27853, *Klebsiella aerogenes* ATCC 13048, and *Shigella boydii* ATCC 8700) tested in this study were clinical isolates obtained from Institute of Medical Sciences, Banaras Hindu University, India. All the bacterial isolates were preliminarily tested for their susceptibility towards different antibiotics. *E. coli* was equally sensitive to chloramphenicol and ampicillin. Though tetracycline was less sensitive, it retained better efficacy at lower concentrations. *Pseudomonas aeruginosa* showed an unexpected sensitivity [44] to ampicillin, while demonstrating moderate sensitivity to chloramphenicol and tetracycline. Non-MRSA *Staphylococcus aureus* was highly sensitive to ampicillin and moderately sensitive to chloramphenicol and tetracycline at high concentrations. This aligns with typical clinical patterns, where non-MRSA strains are generally susceptible to β-lactams and other antibiotics. *Staphylococcus aureus* MRSA was completely resistant to ampicillin and chloramphenicol but sensitive towards tetracycline at highest concentrations only. *Klebsiella aerogenes* was less sensitive towards ampicillin and tetracycline, and moderately sensitive to chloramphenicol at high concentrations, which was consistent with the known resistance mechanisms of *Klebsiella*, including β-lactamase production, efflux pumps, enzymatic inactivation, and biofilm formation [45]. *Shigella boydii* showed low sensitivity to ampicillin and tetracycline, with moderate sensitivity to chloramphenicol at high concentrations. *Pseudomonas azotoformans* showed moderate sensitivity to ampicillin and chloramphenicol at high concentrations and low sensitivity to tetracycline. *Enterobacter cloacae* showed high resistance to ampicillin, chloramphenicol, and tetracycline, aligning with its intrinsic and acquired resistance mechanisms. *Burkholderia paludis* showed low sensitivity to ampicillin and tetracycline and moderate sensitivity to chloramphenicol at high concentrations. All three selected antibiotics showed reduced effectiveness as their concentrations were decreased for all the selected bacterial isolates.

**Figure 12:**
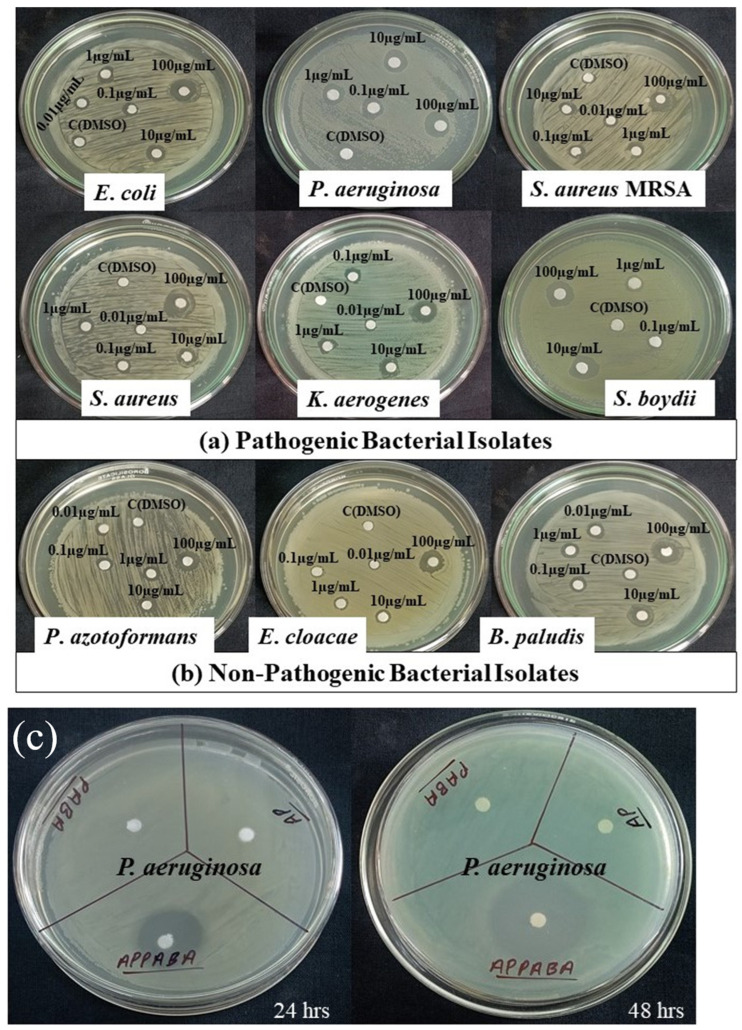
Screening of APPABA for its antibacterial activity based on the inhibition zones formed against different bacterial isolates: (**a**) pathogenic bacteria and (**b**) non-pathogenic bacteria. (**c**) Screening antibacterial activity of parent and synthesized compounds against *P. aeruginosa* at different time intervals.

**Figure 13:**
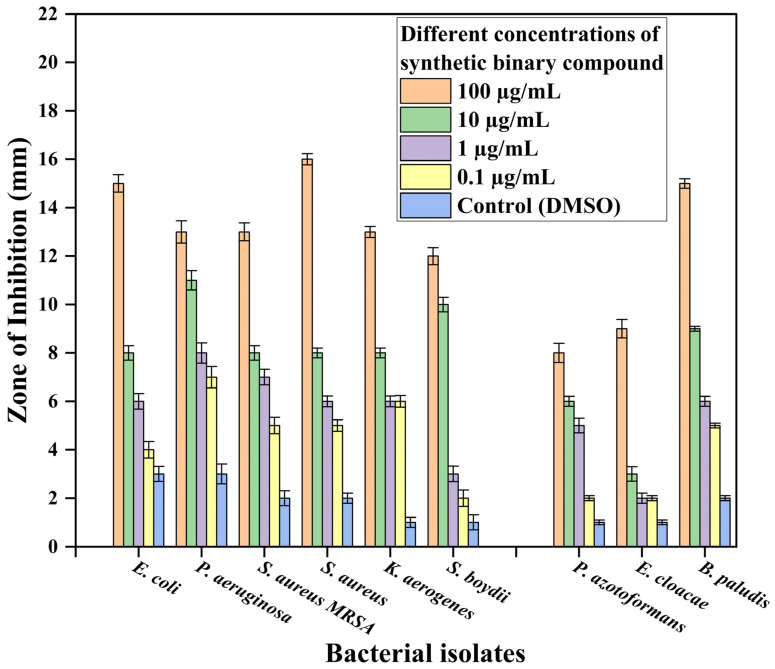
Bar graph showing the zone of inhibition formed by the synthesized binary compound (APPABA) against several pathogenic and non-pathogenic bacterial isolates to determine the minimum inhibitory concentration (MIC) using the disc diffusion method. The means of three replicates were used to compare treatments using Tukey’s multiple range test at *p* ≤ 0.05; the slanting bars indicate the standard error of the mean.

Compared to antibiotics the minimum inhibitory concentration (MIC) of APPABA measured through the disc diffusion method was 0.1 µg/mL for all the selected bacterial strains. Among the various pathogenic bacterial isolates tested, the inhibition zone formed by APPABA was maximum against *P. aeruginosa* (9.75 mm), followed by *S. aureus* (8.75 mm), *E. coli* (8.25 mm), the methicillin-resistant bacterial strain viz. *S. aureus* MRSA (8.25 mm), *K. aerogenes* (8.25 mm), and *S. boydii* (6.75 mm). These results are shown in Figure 12 and Figure 13, with the minimum inhibitory concentration (MIC) being 0.1 µg/mL. The compound APPABA also exhibited good antibacterial activity against the non-pathogenic bacterial isolates, showing a maximum zone of inhibition around *B. paludis* (8.75 mm), followed by *P. azotoformans* (5.25 mm) and *E. cloacae* (4.0 mm), with the minimum inhibitory concentration (MIC) being 0.1 µg/mL. The parent compounds, AP and PABA, screened for antibacterial activity, showed negative results, as no inhibition zone was observed against the tested bacterium *Pseudomonas aeruginosa*, as shown in Figures S8 and S9 in the Supplementary Information. Even after increasing the incubation period from 24 to 48 h, no considerable zone of inhibition was observed against the bacterial lawn for the parent compounds. At the same time, the zone of inhibition formed by the synthesized compound APPABA increased from 14 mm to 25 mm after extending the incubation period from 24 h to 48 h, as depicted in Figure 12c.

The effect of the antibiotics chloramphenicol, ampicillin, and tetracycline on bacterial isolates is presented in Figures S10–S13 in the Supplementary Information and Table 5, respectively. The concentration of these antibiotics significantly impacted (*p* < 0.001) the zone of inhibition against pathogenic bacterial isolates. As the concentration decreased, the zones of inhibition also decreased. Chloramphenicol had the most significant effect on *E. coli* and *P. aeruginosa* (6.75 mm), followed by *S. aureus* (5.75 mm), *K. aerogenes*, and *S. boydii* (4.5 mm), *P. azotoformans* and *E. cloacae* (2.25 mm), *B. paludis* (1.75 mm), and *S. aureus MRSA* (0.3 mm), as presented in Figure S10 in the Supplementary Information, with the minimum inhibitory concentration (MIC) being 0.1 µg/mL. Ampicillin had the most significant effect on *P. aeruginosa* (9.0 mm), followed by *S. aureus* (8.75 mm), *E. coli* (6.75 mm), *K. aerogenes* (3.5 mm), *S. boydii* (2.5 mm), *P. azotoformans* (3.0 mm), *E. cloacae* (1.7 mm), and *B. paludis* (1.5 mm), as presented in Figure S11 in the Supplementary Information, with the minimum inhibitory concentration (MIC) being 0.1 µg/mL. Tetracycline had the most significant effect on *E. coli* and *S. aureus MRSA* (7.75 mm), followed by *P. aeruginosa* (7.25 mm), *S. aureus* (6.5 mm), *K. aerogenes* (2.75 mm), *S. boydii* (2.5 mm), *P. azotoformans* (2.25 mm), *B. paludis* (1.0 mm), and *E. cloacae* (0.75 mm), as presented in Figure S12 in the Supplementary Information, with the minimum inhibitory concentration (MIC) being 0.1 µg/mL.

The results obtained showed that the synthesized compound (APPABA) had the highest antibacterial activity against all the pathogenic and non-pathogenic bacterial isolates, while the tested antibiotics (chloramphenicol, ampicillin, and tetracycline) were less active compared to the synthesized compound. These results confirm that the synthesized compound can be used alongside conventional antibiotics to combat infectious agents. This study demonstrates the potency of the synthesized compound (APPABA) and the selected antibiotics (chloramphenicol, tetracycline, and ampicillin) against bacterial isolates. The large zones of growth inhibition formed by APPABA and the antibiotics suggest that the active constituents in the parent compounds (AP and PABA), as well as those in the antibiotics, are similarly effective. The results confirm the therapeutic potential of natural alternatives to pharmaceutical antibiotics. This study consistently demonstrated the effectiveness of the synthesized compound as an antibacterial agent against both pathogenic and non-pathogenic bacteria.

Additionally, the absorption, distribution, metabolism, excretion, and toxicity (ADMET) data are presented in Table S2 (Supplementary Information). The toxicity findings show no AMES toxicity, indicating that the compound is likely non-carcinogenic. It does not inhibit hERG I or hERG II, and the maximum tolerated dose for humans is 0.586 log mg/kg/day. The results indicate that the molecule is not harmful upon skin contact; however, it is hepatotoxic.

Moreover, the data suggests that the compound has moderate intestinal absorption but poor skin permeability. Its lack of interaction with P-glycoproteins implies that it may not significantly influence the absorption of other substances. The compound shows limited distribution and permeability to the central nervous system, does not interact with major metabolic enzymes, and has a slow clearance rate from the body. Additionally, it is not a substrate for renal OCT2, indicating minimal renal excretion via this pathway.

## 3. Materials and Methods

### 3.1. Materials and Purification

The parent compounds used in the present study, 2-aminopyrimidine (AP) and 4-aminobenzoic acid (PABA), were procured from Sigma-Aldrich (Darmstadt, Germany) and Alfa Aesar by Thermo Fisher Scientific (Mumbai, India), respectively. The purity of both parent compounds was verified by their melting points and NMR studies and were found to be in agreement with the values reported in the literature [46].

### 3.2. Phase Diagram Study

The phase diagrams represent the melting temperature–composition curve [47]. To establish the phase diagram of the AP—PABA binary system, the mixtures of the AP and PABA compounds from 0 to 1 mole fractions were prepared by weighing their appropriate amount using the physical balance (Denver SI-234, Denver Instrument Company, Denver, CO, USA) of accuracy ± 0.0001 g. These mixtures were placed in different dried test tubes, and the mouth of each test tube was sealed. Then, each sealed test tube was placed individually in a pre-heated silicon oil bath, and the mixtures were melted and homogenized by repeating the process of melting/mixing, followed by chilling in ice-cold water 4 times. The test tube, containing the homogenized mixture, was broken, and the solid mixture was ground using a mortar and pestle, and the melting point of each compositional mixture was recorded using the Toshniwal melting point apparatus, equipped with a thermometer, which can read measurements with an accuracy of 0.5 degrees Celsius. The melting points of IMC and eutectics were further confirmed by differential scanning calorimeters. The phase diagram of PA—PABA was plotted by taking the mole fractions of PA on the *X*-axis and their corresponding melting temperatures on the Y-axes.

### 3.3. Thermal Study

To determine the experimental values of the heat of fusion values and to study the various thermal properties of the AP—PABA system, a pre-calibrated differential scanning calorimeter (DSC) (Mettler DSC-4000 system) using indium and zinc samples was used. The melting temperature and thermal behaviour of the parent compounds, eutectics and IMC (APPABA) were studied by taking 4–6 mg of samples and keeping a heating rate of 10 °C/min under the constant flow (20 mL/min) of nitrogen gas. The values of heat of fusion were found to be reproducible within ±0.01 kJ/mol.

### 3.4. Spectral Study

To elucidate the structure of the synthesized IMC, the FTIR and NMR spectral techniques were employed. The FTIR spectra of the parent compounds and the IMC were recorded using a Perkin Elmer spectrophotometer (PerkinElmer, Waltham, MA, USA) by dispersing and pelletizing the powder samples in KBr. However, the proton and carbon spectra of the synthesized intermolecular compound along with parent compounds were recorded using JNM-ECZ500R/S1 500 MHz Spectrometer (JEOL Ltd., Tokyo, Japan) in DMSO-d6 solvent. The mass spectrum of the parent compounds and synthesized APPABA were recorded using the SCIEX X500R QTOF (TOF-MS) instrument (SCIEX, Toronto, ON, Canada).

### 3.5. Powder X-Ray Diffraction Study

The X-ray diffraction analysis of the parent compounds, their eutectics, and the intermolecular compound were recorded using a Rigaku powder diffractometer (Rigaku Corp., Tokyo, Japan) equipped with an 18-kW rotating copper anode and a graphite monochromator. The samples were recorded at a scanning rate of 3°/min.

### 3.6. Single-Crystal Growth and X-Ray Diffraction Study

The single crystals of the novel APPABA intermolecular compound were grown in a mixed solvent, ethanol/water (in a 3:2 ratio), adopting the slow solvent evaporation method; however, the temperature of the solution was maintained in the temperature-controlled water bath. The tiny crystals that appeared were allowed to grow for 13 days. The single-crystal X-ray data was collected using a Bruker single-crystal X-ray diffractometer (Billerica, MA, USA), and the refinement was performed using the Olex 2_1.2 program suite [48]. The structure was solved using SHELXT [49] and refined using SHELXL [50]. The crystal packing diagrams were generated using the Mercury 4.0 program [51]. The CIF structure file has been deposited in the CCDC database, and the allotted CCDC is 2235290.

### 3.7. Optical Study

The UV–visible absorption spectrum of IMC, along with the parent compounds, was recorded by preparing dilute solutions of uniform concentration (1 × 10^−5^ M) at room temperature (300 K) and analysing them using a UV/Vis/NIR spectrometer (JASCO model V-670, Tokyo, Japan). To record the fluorescence properties of the respective sample solutions at the same temperature, the Varian Cary Eclipse fluorescence spectrometer was used. The influence of solvent polarity was also studied using the solvent of variable polarity and the same spectrometer, under identical conditions.

### 3.8. Antibacterial Studies

The antibacterial potency of the synthesized IMC and antibiotics were examined based on their inhibitory activity against the pathogenic bacterial isolates: *Escherichia coli*, *Pseudomonas aeruginosa*, *Staphylococcus aureus*, methicillin-resistant *Staphylococcus aureus* (MRSA), *Klebsiella aerogenes*, and *Shigella boydii*; non-pathogenic bacterial isolates: *Enterobacter cloacae*, *Pseudomonas azotoformans*, and *Burkholderia paludis*, following the Kirby–Bauer disc diffusion method, at different concentrations. The different bacterial strains were inoculated separately on a yeast extract mannitol agar (YEMA) medium containing, per litre of distilled water: yeast extract, 1.00 g; KH_2_PO_4_, 0.50 g; mannitol, 10.00 g; MgSO_4_·7H_2_O, 0.20 g; NaCl, 0.10 g; and agar, 15.00 g, adjusting the pH to 6.8–7.0. The different concentrations (100, 10, 1, and 0.1 µg/mL in DMSO) of antibiotics (chloramphenicol, ampicillin, and tetracycline) and the synthesized compound were obtained through serial dilutions. Bacteria were picked from sub-cultures and inoculated into YEMA plates through the spread plate method. Discs containing different antibiotics and the synthesized intermolecular compound were kept on each Petri plate and incubated at 28 °C for 24 h, followed by measuring the inhibition zone. The control was maintained using discs dipped only in DMSO. The parent compounds (AP, PABA) were also screened (0.1 mg/mL in DMSO) to assess their inhibitory effects against *Pseudomonas aeruginosa* at different time intervals. Software pkCSM (https://biosig.lab.uq.edu.au/pkcsm/prediction, accessed on 3 May 2025) was used to predict pharmacokinetic properties, including absorption, distribution, metabolism, excretion, and toxicity (ADMET) [52].

## 4. Conclusions

In this work, 2-aminopyrimidine and para-aminobenzoic acid were utilized to synthesize a novel binary organic material. After the synthesis of IMC, crystals were grown, and characterizations were performed, including NMR, FTIR, PXRD, DSC, HRMS, and single-crystal XRD. Hirshfeld surface analysis was conducted to understand the interactions, and melting temperatures were used for thermodynamic analysis. Finally, an antimicrobial study was carried out, revealing that the synthesized material exhibits antimicrobial properties. Based on these studies, the following conclusions have been drawn:

(1) The solid-state green synthesis approach employed for the synthesis of APPABA offers several significant advantages compared to other synthesis methods. This approach is environmentally friendly, eliminates the use of solvents, is less complex, time-efficient, and cost-effective compared to other organic synthesis methods. The use of only melting temperatures for synthesis, avoiding solvents and complexity, enhances its practicality and accessibility in both academic and industrial applications.
(2) The compositional behaviour as a function of melting temperature has been studied by establishing the solid–liquid phase equilibrium diagram, which suggests the formation of a 1:1 intermolecular compound (IMC) and two eutectics. The DSC studies confirmed the purity and single-phase transition of the IMC during melting.
(3) Spectral studies of the IMC confirmed the formation of hydrogen bonds, showing that the two molecules are joined together via hydrogen bonding between the carboxylic group of PABA and both the amine group and the ring nitrogen atom of AP. The optical studies suggest that the IMC (APPABA) is significantly fluorescent. Additionally, the single-crystal X-ray diffraction study of the grown crystal of IMC confirms its crystal structure to be monoclinic with a P21/n space group.
(4) The antibacterial activity of APPABA, tested through inhibition zone assays, showed a range of 6.75–9.75 mm for pathogenic strains and 4–8.75 mm for non-pathogenic strains. APPABA was particularly effective in inhibiting the methicillin-resistant bacterial strain *Staphylococcus aureus* (MRSA). The MIC of APPABA against all the selected bacterial strains was 0.1 µg/mL, which was comparable to the selected antibiotics. Furthermore, an expanding antibacterial zone, from 14 mm to 25 mm, was observed over time against the pathogenic *Pseudomonas aeruginosa*.

Overall, this study represents the green synthesis of an intermolecular compound. The spectral studies confirmed the structure; additionally, the thermodynamic, antimicrobial, and crystal structure have been investigated. These studies and observations suggest that this synthesized biodegradable compound, APPABA, is an effective functional material with potential for future application as an antibacterial agent.

## Figures and Tables

**Table 1:** Crystal data and structure refinement of the APPABA intermolecular compound.

	APPABA
Empirical formula	C_22_H_24_N_8_O_4_
T/K	296
Crystal System	Monoclinic
Space Group	*P*21/n
a/Å	5.2428 (2)
b/Å	17.9260 (5)
c/Å	11.8917 (4)
α/°	90
β/°	92.5750 (10)
γ/°	90
V/Å^3^	2416.5 (2)
Z	2
μ (Mo−Kα)/mm^−1^	0.099
Reflections Collected/unique	187.36/2754
R (int)	0.0381
Final R indices [I > 2σ (I)]	R1 = 0.0440 wR2 = 0.1143
R indices (all data)	R1 = 0.0554 wR2 = 0.1214
GOF on F2	1.060
CCDC No	2235290

**Table 2:** Melting temperature, heat of fusion, heat of mixing, and entropy of fusion of the AP—PABA system.

Component	Melting Temperature (K)	Heat of Fusion (kJ mol^−1^)	Heat of Mixing (kJ mol^−1^)	Entropy of Fusion (kJ mol^−1^ K^−1^)
*AP—PABA system*				
AP	398.41	18.32		0.0460
PABA	462.58	20.73		0.0448
Eutectic-1	429.00			
(exp.)		26.95	−0.43	0.0628
(cal.)		27.38		
Eutectic-2	393.62			
(exp.)		22.06	−4.11	0.0560
(cal.)		26.17		
APPABA (1:1)	439.88	34.03		0.0774

**Table 3:** Roughness parameter (α), interfacial energy (σ), and grain boundary energy (γ) of AP, PABA, eutectics, and the (1:1) APPABA intermolecular compound.

Component	α	σ (erg cm^−2^)	γ (erg cm^−2^)
*AP—PABA system*			
AP	5.53	38.91	77.83
PABA	8.38	39.92	79.85
Eutectic-1	7.55	39.67	79.34
Eutectic-2	6.73	39.82	79.65
APPABA (1:1)	9.35	39.42	78.84

**Table 4:** Excess thermodynamic functions for the eutectics of AP—PABA system.

Component	g^E^ (kJ mol^−1^)	h^E^ (kJ mol^−1^)	s^E^ (J mol^−1^K^−1^)
*AP—PABA system*			
Eutectic-1	0.36	6.73	0.0149
Eutectic-2	1.22	4.02	0.0071

**Table 5:** Zone of inhibition of various bacterial isolates to determine the MIC of synthesized compound and antibiotics.

Concentrations	Synthesized Compound and Antibiotics	*E. coli*	*P. aeruginosa*	*S. aureus* MRSA	*S. aureus*	*K. aerogenes*	*S. boydii*	*P. azotoformans*	*E. cloacae*	*B. paludis*
**100 µg/mL**	APPABA	15	13	16	13	13	12	8	9	15
	Chloramphenicol	13	10	0.3	8	7	7	6	6	6
	Ampicillin	13	14	0	14	6	4	10	4	4
	Tetracycline	12	9	18.4	11	6	4	5	2	3
**10 µg/mL**	APPABA	8	11	8	8	8	10	6	3	9
	Chloramphenicol	8	7	0	6	5	5	2	2	1
	Ampicillin	9	10	0	10	4	3	3	2	1
	Tetracycline	8	8	12.4	8	3	3	3	1	1
**1 µg/mL**	APPABA	6	8	6	7	6	3	5	2	6
	Chloramphenicol	3	6	0	5	4	4	1	1	0
	Ampicillin	3	7	0	6	3	2	2	1	1
	Tetracycline	6	7	0.2	5	1	2	1	0	0
**0.1 µg/mL**	APPABA	4	7	5	5	6	2	2	2	5
	Chloramphenicol	3	4	0	4	2	2	0	0	0
	Ampicillin	2	5	0	5	1	2	0	0	0
	Tetracycline	5	5	0	2	1	1	0	0	0
**Control**	APPABA	3	3	2	2	1	1	1	1	2
	Chloramphenicol	2	3	0.1	2	2	2	0.5	0.7	0.5
	Ampicillin	2	3	0	3	1	1	0.7	0.5	0.5
	Tetracycline	3	2	0.1	2	0.6	0.8	0.7	0.6	0.5

## Data Availability

Data will be made available on request.

## Supplementary Materials

The following supporting information can be downloaded at https://www.mdpi.com/article/10.3390/ijms26125509/s1.
