# Supplementary material for: Green Solid-State Synthesis of Antibacterial Binary Organic Material: Crystal Growth, Physicochemical Properties, Thermal Study, Antibacterial Activity, and Hirshfeld Surface Analysis
Source: Int J Mol Sci. 2025 Jun 9;26(12):5509. doi: 10.3390/ijms26125509 (PMC12193437; doi:10.3390/ijms26125509)
Supplement: Supplementary file 1 [file ijms-26-05509-s001.zip › ijms-3556002-supplementary.pdf]

Supplementary information

Table S1: Experimental solid–liquid equilibrium data and liquid mole fractions X for the AP—PABA system.

S. No.	AP—PABA System		
	Mole fraction (X_{AP})	Melting temperature (°C)	Solid phase
1	0.00	189.4	PABA
2	0.10	172.0	PABA
3	0.15	166.0	PABA
4	0.20	161.0	PABA
5	0.23	157.0	PABA
6	0.25	155.8	PABA+APPABA
7	0.28	157.0	APPABA
8	0.30	158.0	APPABA
9	0.40	163.0	APPABA
10	0.48	166.0	APPABA
11	0.50	166.7	APPABA
12	0.52	165.0	APPABA
13	0.60	160.0	APPABA
14	0.70	153.0	APPABA
15	0.80	143.0	APPABA
16	0.85	136.0	APPABA
17	0.88	123.0	APPABA
18	0.90	120.4	APPABA+AP
19	0.92	123.0	AP
20	1.00	125.2	AP

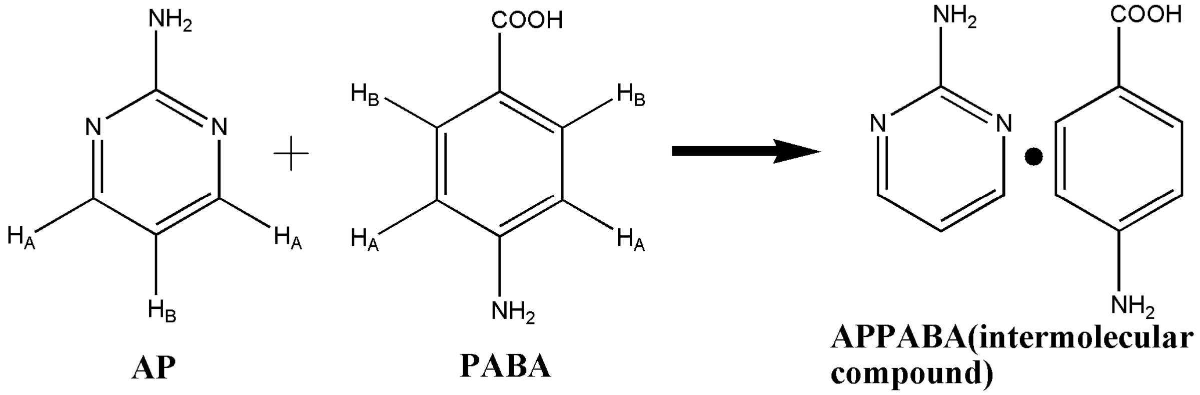

AP **PABA** **APPABA(intermolecular compound)**

Figure S1: Solid state reaction scheme between AP and APPABA resulting into the formation of the APPABA intermolecular compound.

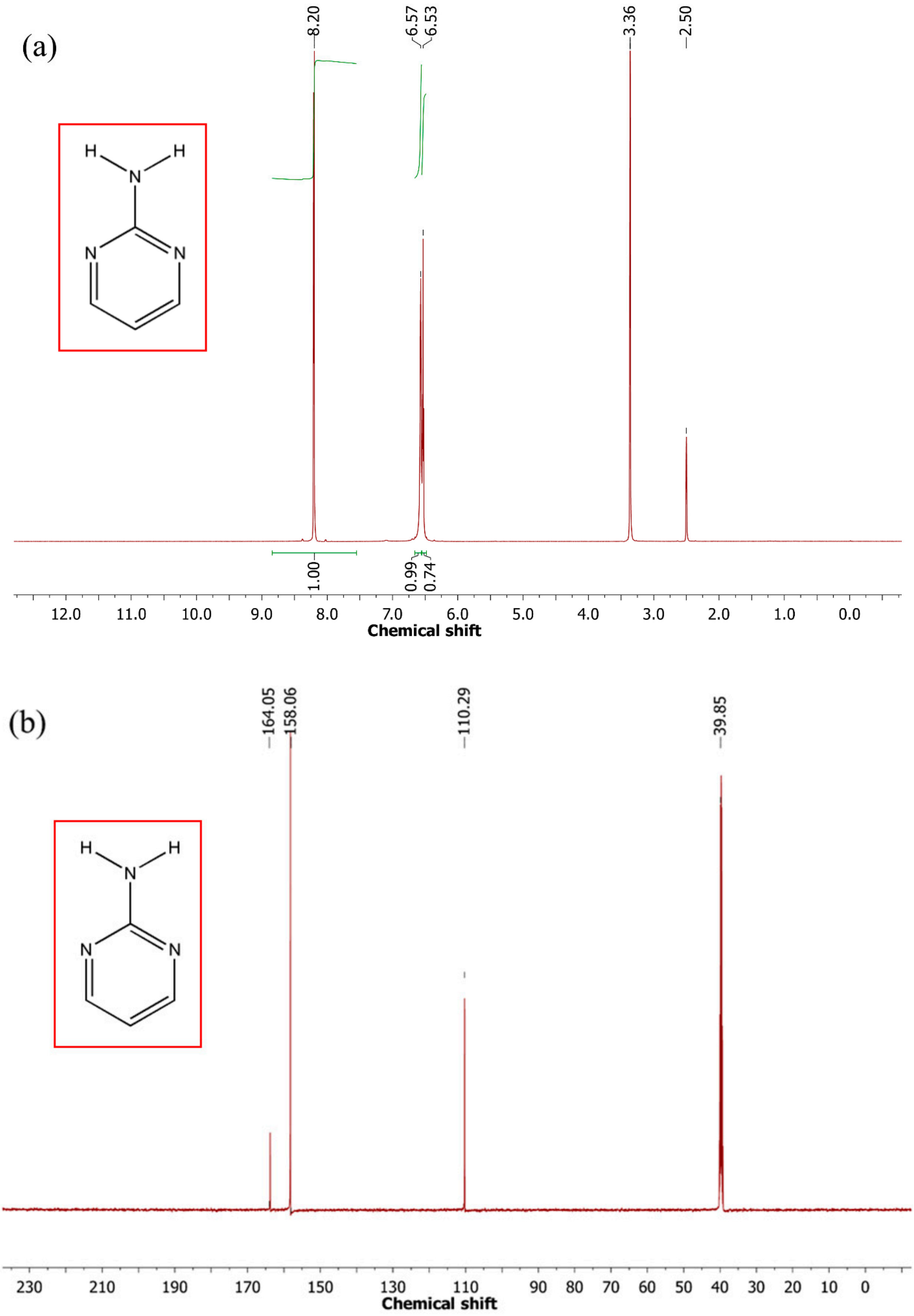

Figure S2: (a) ^{1}H NMR and (b) ^{13}C NMR of 2-aminopyrimidine (AP).

(a)

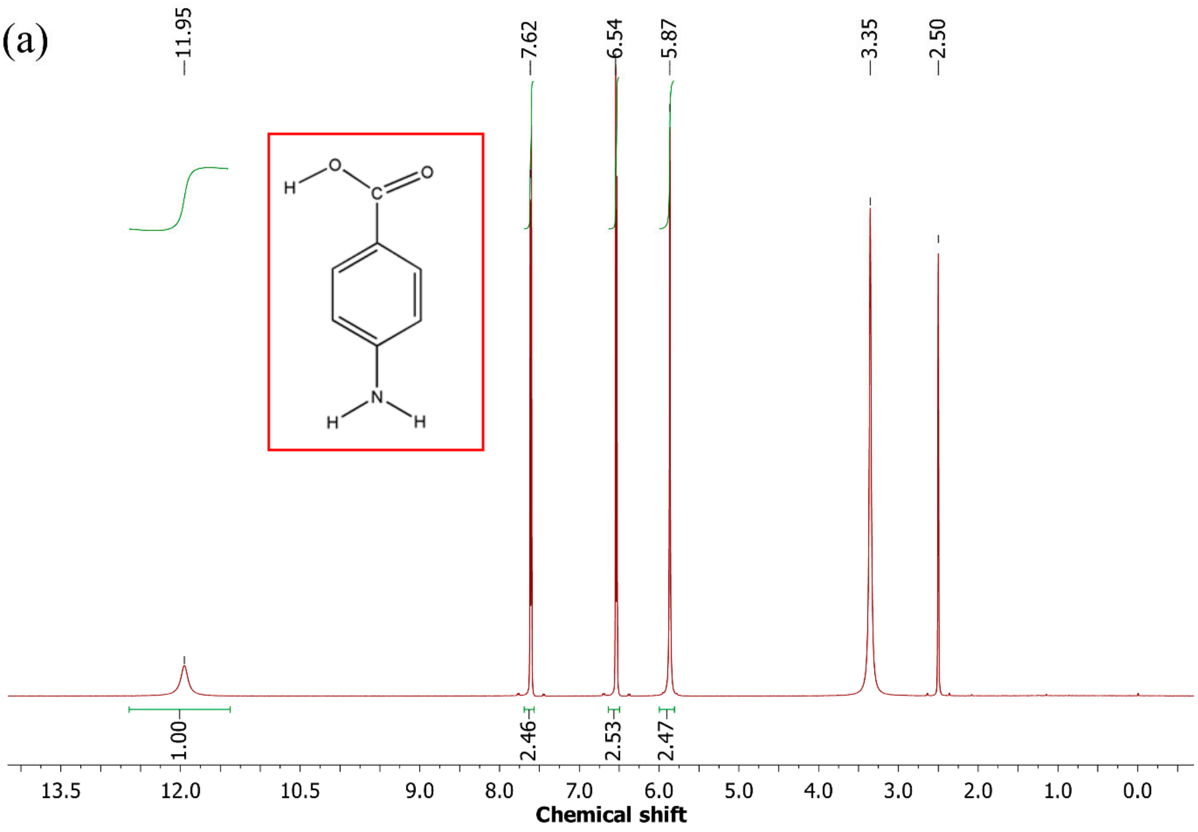

(b)

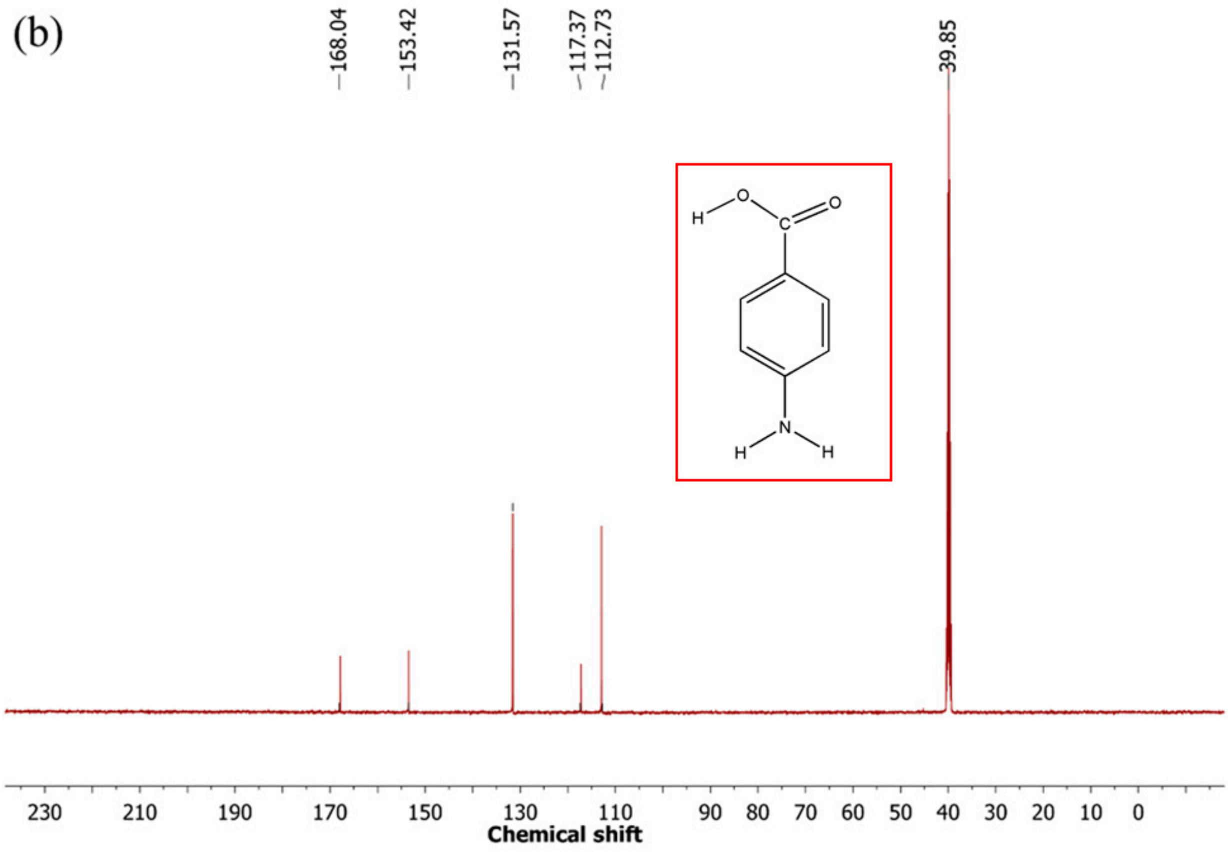

Figure S3: (a) ^{1}H NMR and (b) ^{13}C NMR of 4-aminobenzoic acid (PABA).

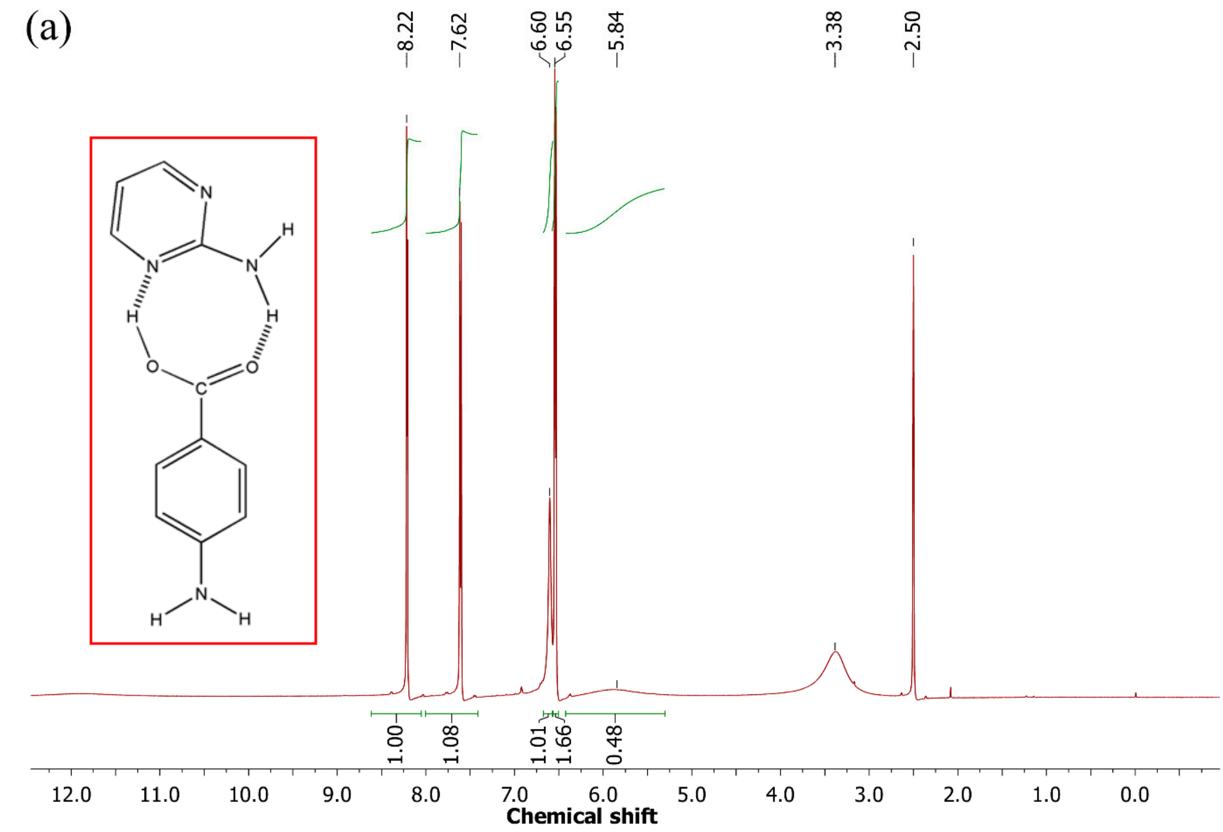

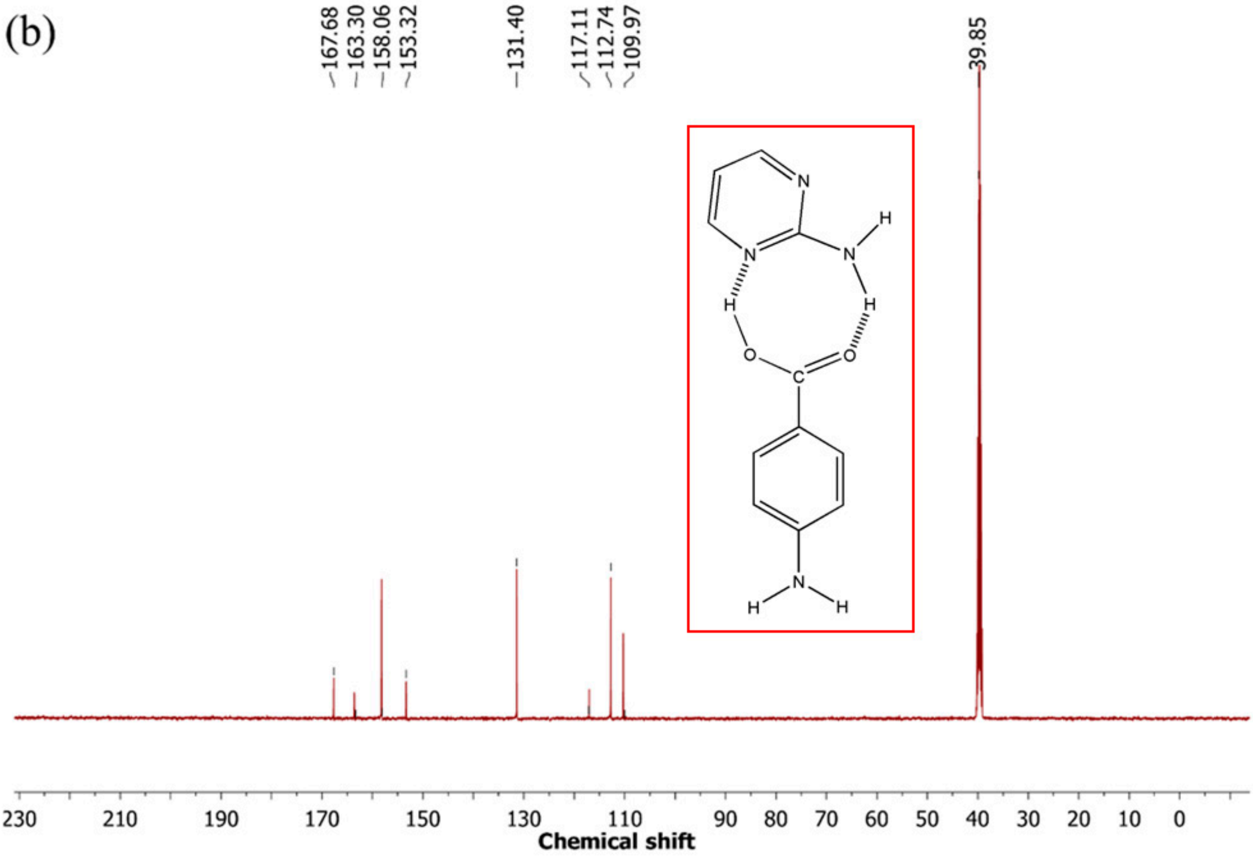

Figure S4: (a) ^{1}H NMR and (b) ^{13}C NMR of the intermolecular compound (APPABA).

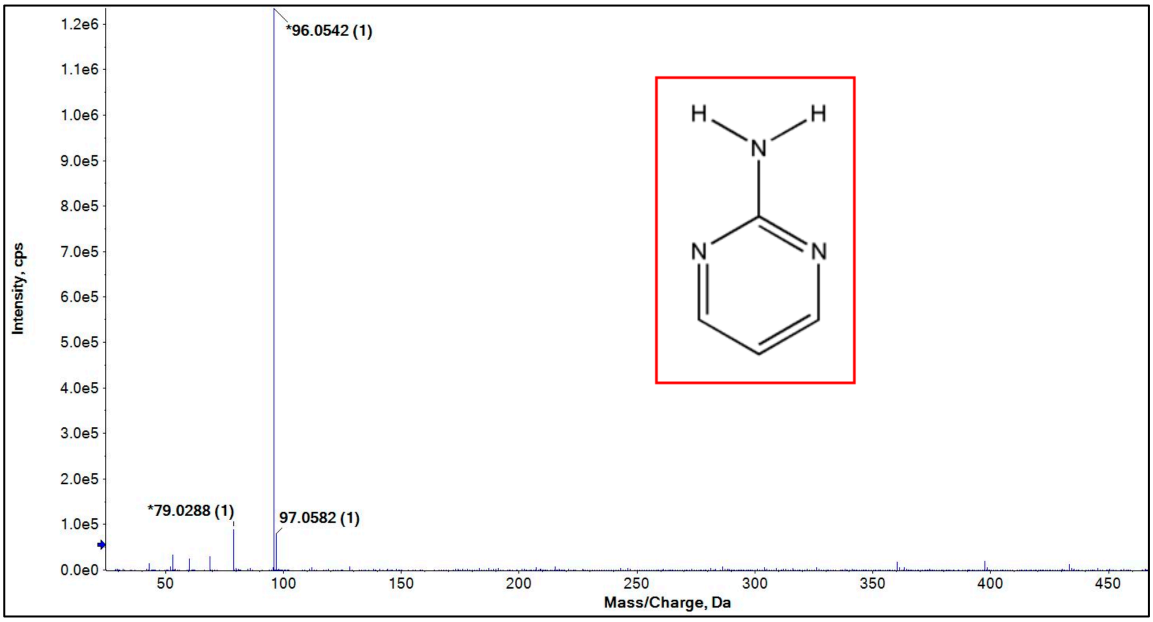

Figure S5: Mass spectrum of the parent compound AP.

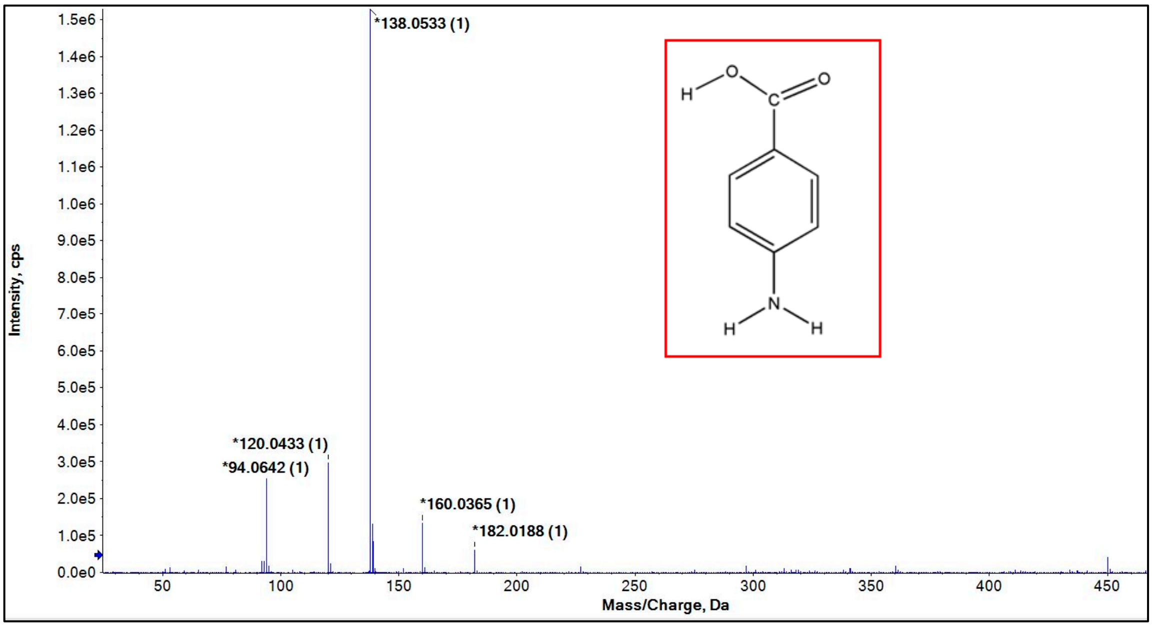

Figure S6: Mass spectrum of the parent compound PABA.

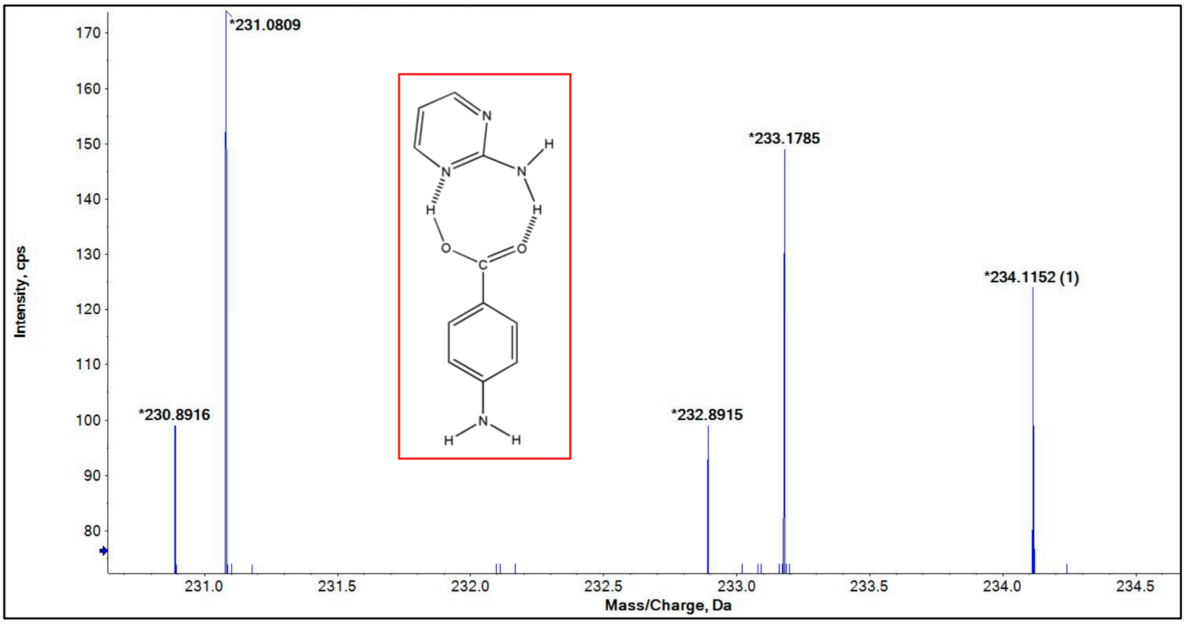

Figure S7: Mass spectrum of the synthesized novel compound APPABA.

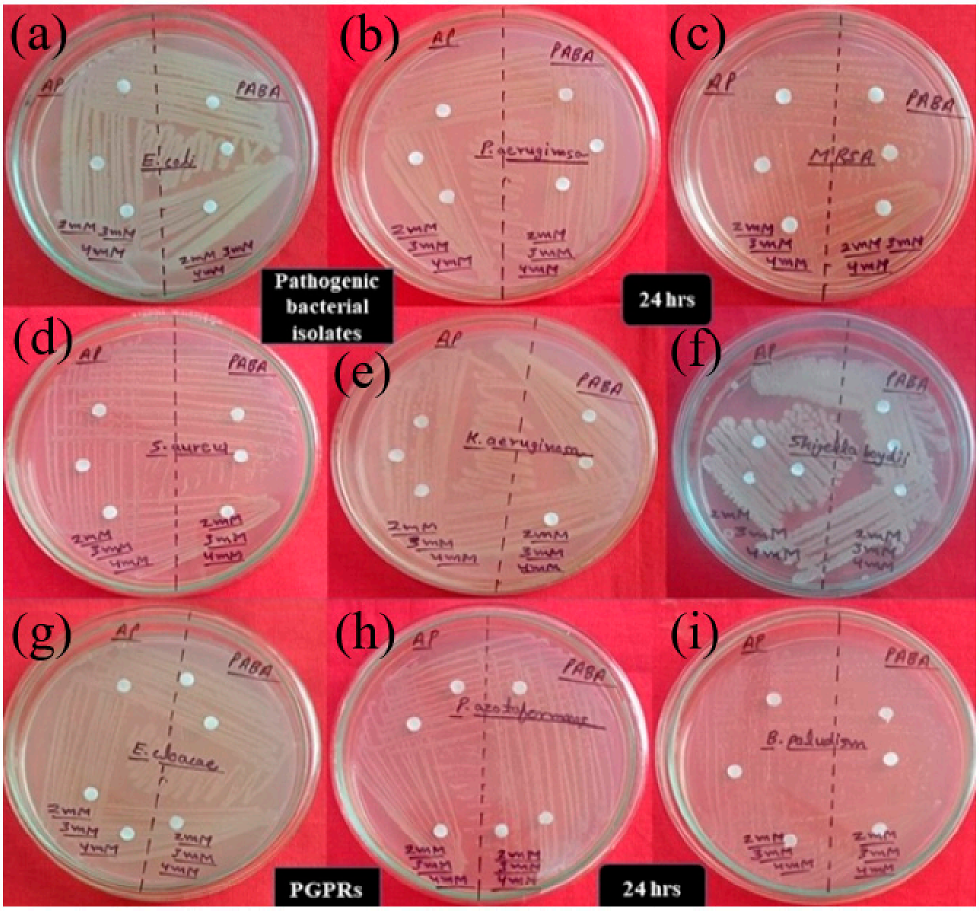

Figure S8: Parent compounds are inactive against the pathogenic bacterial isolates and PGPRs and hence do not exhibit the zone of inhibition.

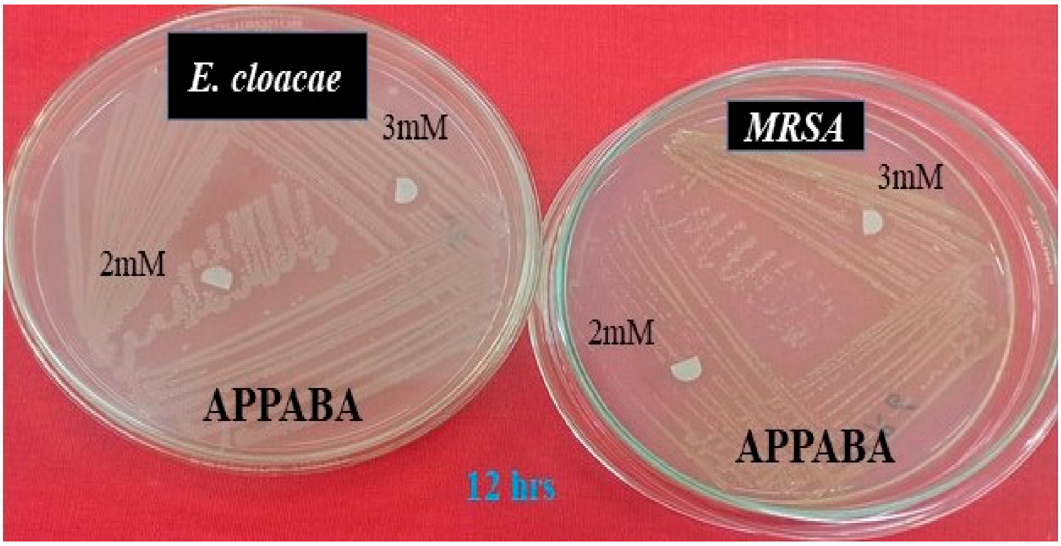

Figure S9: Compound APPABA exhibiting zone of inhibition on bacterial streaking.

Table S2: Results of ADMET analysis of APPABA using the pkCSM online server.

**Property**	**Model Name**	**Predicted Value**	**Unit**
**Absorption**	Water solubility	-2.394	Numeric (log mol/L)
**Absorption**	Caco2 permeability	-0.122	Numeric (log Papp in 10-6 cm/s)
**Absorption**	Intestinal absorption (human)	56.939	Numeric (% Absorbed)
**Absorption**	Skin Permeability	-2.735	Numeric (log Kp)
**Absorption**	P-glycoprotein substrate	No	Categorical (Yes/No)
**Absorption**	P-glycoprotein I inhibitor	No	Categorical (Yes/No)
**Absorption**	P-glycoprotein II inhibitor	No	Categorical (Yes/No)
**Distribution**	VDss (human)	-1.599	Numeric (log L/kg)
**Distribution**	Fraction unbound (human)	0.448	Numeric (Fu)
**Distribution**	BBB permeability	-0.871	Numeric (log BB)
**Distribution**	CNS permeability	-2.552	Numeric (log PS)
**Metabolism**	CYP2D6 substrate	No	Categorical (Yes/No)
**Metabolism**	CYP3A4 substrate	No	Categorical (Yes/No)
**Metabolism**	CYP1A2 inhibitior	No	Categorical (Yes/No)
**Metabolism**	CYP2C19 inhibitior	No	Categorical (Yes/No)
**Metabolism**	CYP2C9 inhibitior	No	Categorical (Yes/No)
**Metabolism**	CYP2D6 inhibitior	No	Categorical (Yes/No)
**Metabolism**	CYP3A4 inhibitior	No	Categorical (Yes/No)

**Excretion**	Total Clearance	0.328	Numeric (log ml/min/kg)
**Excretion**	Renal OCT2 substrate	No	Categorical (Yes/No)
**Toxicity**	AMES toxicity	No	Categorical (Yes/No)
**Toxicity**	Max. tolerated dose (human)	0.586	Numeric (log mg/kg/day)
**Toxicity**	hERG I inhibitor	No	Categorical (Yes/No)
**Toxicity**	hERG II inhibitor	No	Categorical (Yes/No)
**Toxicity**	Oral Rat Acute Toxicity (LD50)	2.024	Numeric (mol/kg)
**Toxicity**	Oral Rat Chronic Toxicity (LOAEL)	1.844	Numeric (log mg/kg_bw/day)
**Toxicity**	Hepatotoxicity	Yes	Categorical (Yes/No)
**Toxicity**	Skin Sensitisation	No	Categorical (Yes/No)
**Toxicity**	T.Pyriformis toxicity	0.283	Numeric (log ug/L)
**Toxicity**	Minnow toxicity	2.032	Numeric (log mM)

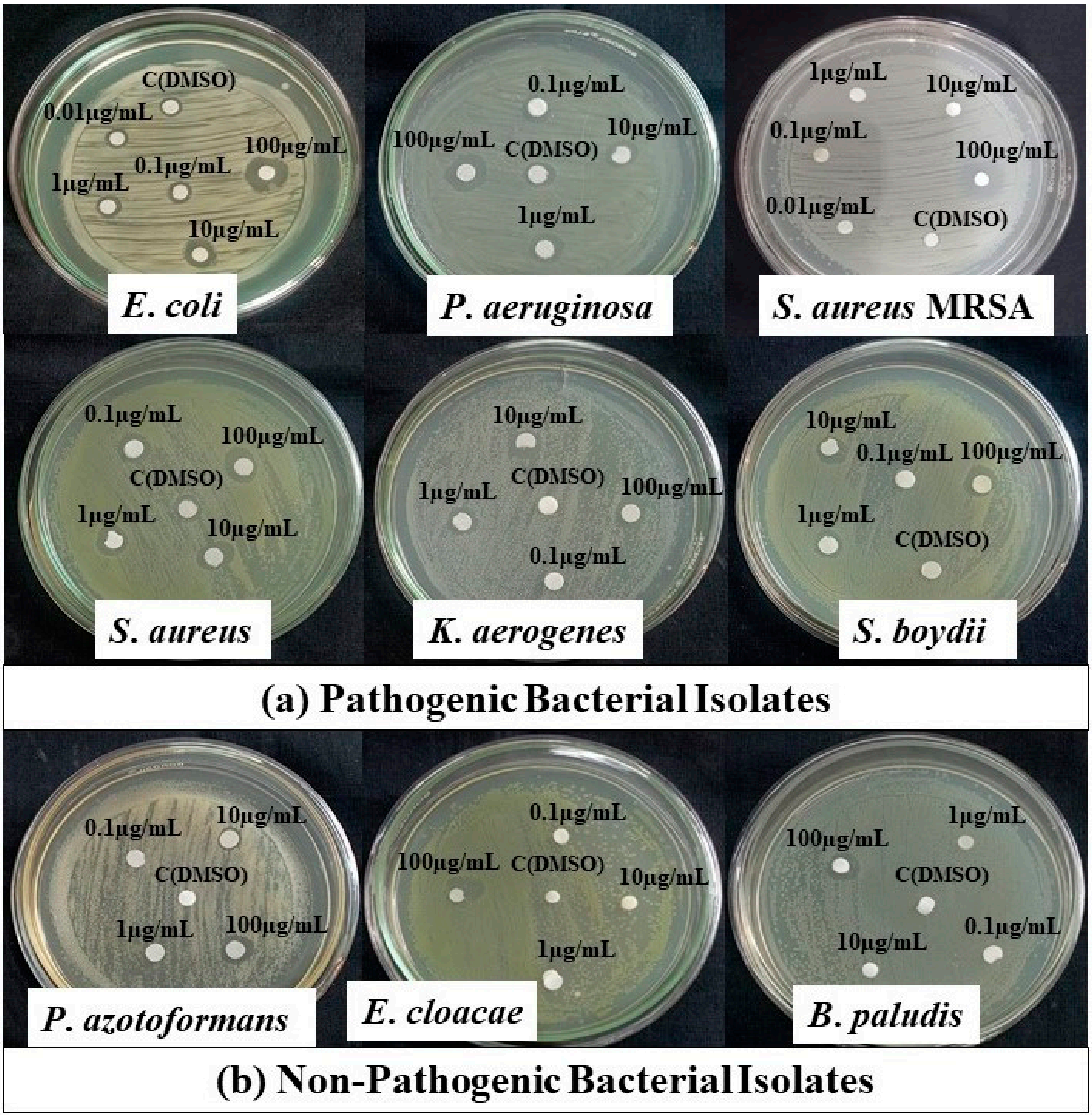

Figure S10: Screening of antibiotic chloramphenicol for its antibacterial activity through the zone of inhibition formed against different bacterial isolates: (a) pathogenic bacteria and (b) non-pathogenic bacteria.

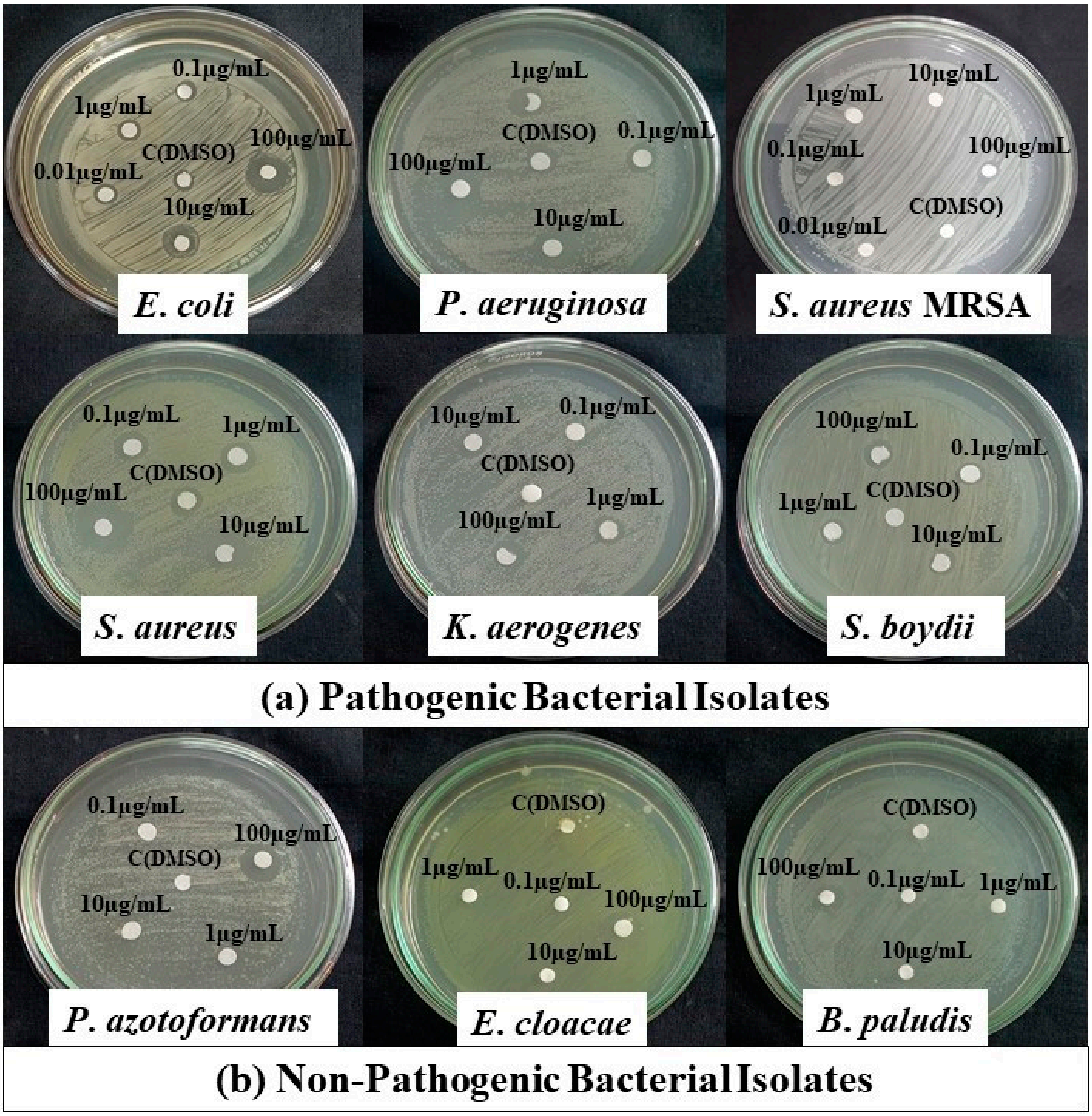

Figure S11: Screening of the antibiotic ampicillin for its antibacterial activity through the zone of inhibition formed against different bacterial isolates: (a) pathogenic bacteria and (b) non-pathogenic bacteria.

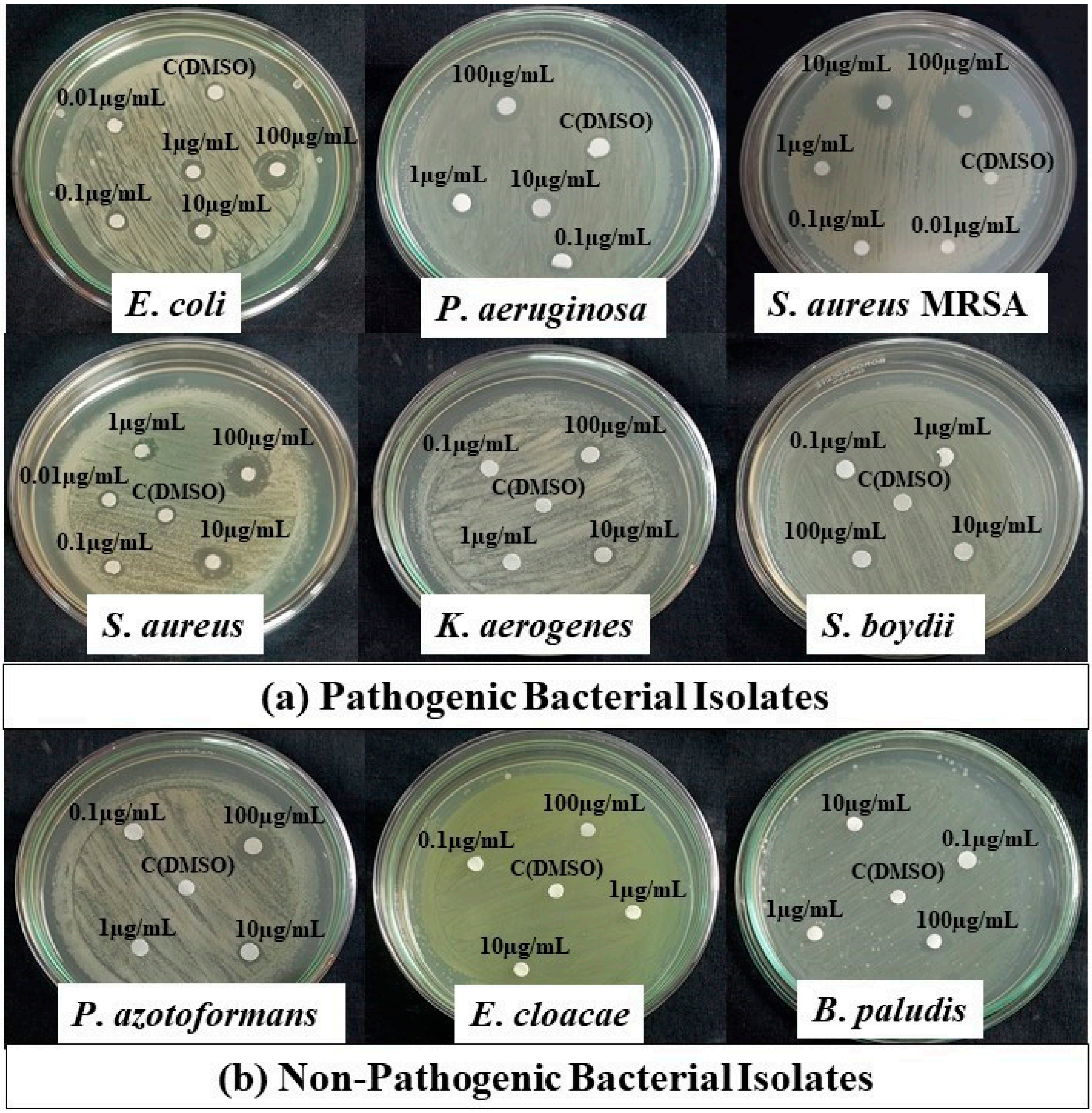

Figure S12: Screening of antibiotic tetracycline for its antibacterial activity through the zone of inhibition formed against different bacterial isolates: (a) pathogenic bacteria and (b) non-pathogenic bacteria.

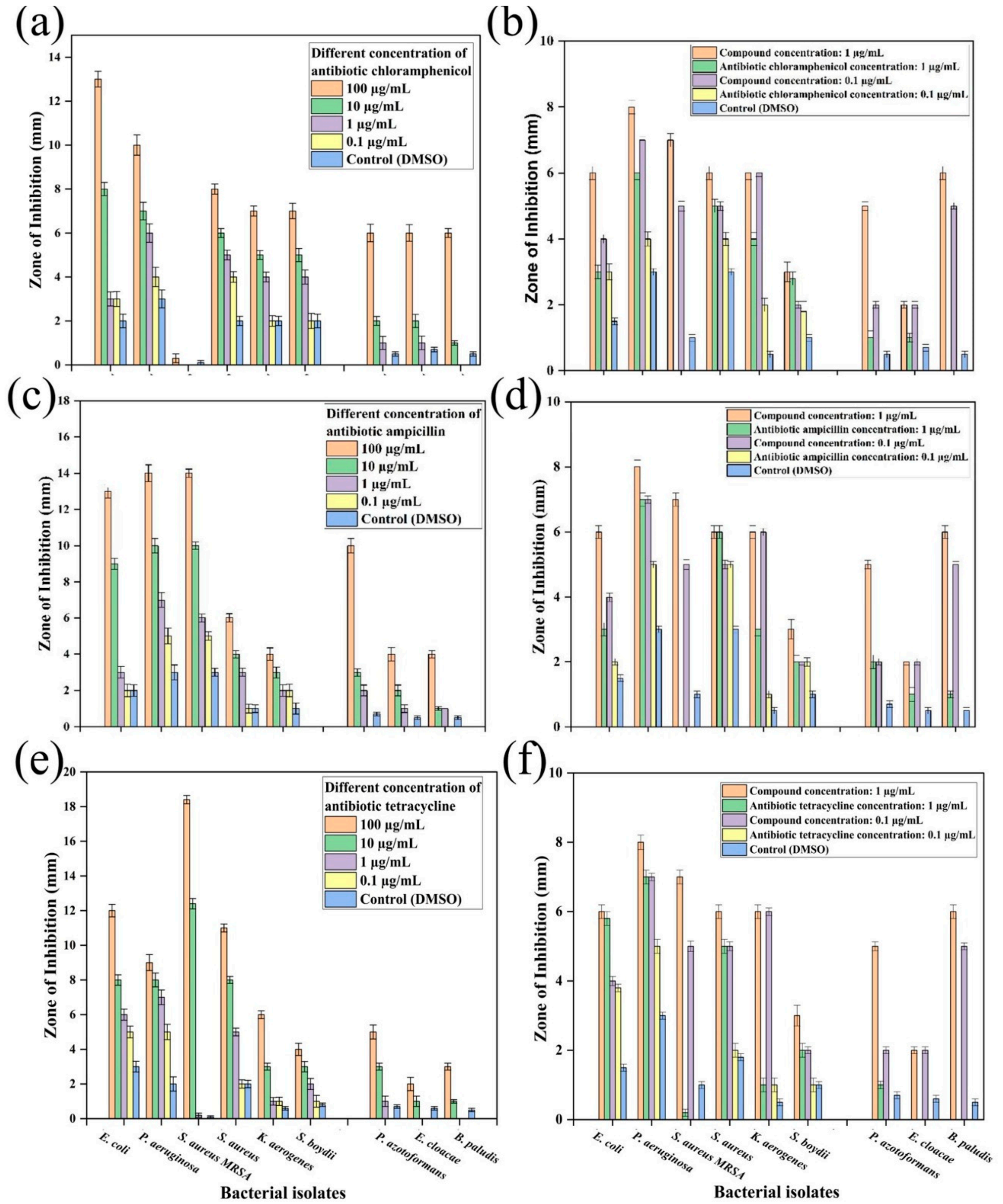

Figure S13. (a) Zone of inhibition for various bacterial isolates to determine the MIC of chloramphenicol; (b) comparison of APPABA and chloramphenicol inhibition; (c) zone of inhibition for MIC of ampicillin; (d) comparison of APPABA and ampicillin inhibition; (e) zone of inhibition for MIC of tetracycline; (f) comparison of APPABA and tetracycline inhibition. The means of three replicates were used to compare treatments using Tukey's multiple range test at $p \leq 0.05$; slanting bars indicate the standard error of the mean.
